# *In silico* Phage Hunting: Bioinformatics Exercises to Identify and Explore Bacteriophage Genomes

**DOI:** 10.3389/fmicb.2020.577634

**Published:** 2020-09-17

**Authors:** Betsy M. Martinez-Vaz, Madeline M. Mickelson

**Affiliations:** Department of Biology, Hamline University, St. Paul, MN, United States

**Keywords:** bacteriophages, bioinformatics, genomes, phylogenetic trees, research project, experimental design (study designs)

## Abstract

Bioinformatics skills are increasingly relevant to research in most areas of the life sciences. The availability of genome sequences and large data sets provide unique opportunities to incorporate bioinformatics exercises into undergraduate microbiology courses. The goal of this project was to develop a teaching module to investigate the abundance and phylogenetic relationships amongst bacteriophages using a set of freely available bioinformatics tools. Computational identification and examination of bacteriophage genomes, followed by phylogenetic analyses, provides opportunities to incorporate core bioinformatics competencies in microbiology courses and enhance students’ bioinformatics skills. The first activity consisted of using PHASTER (PHAge Search Tool Enhanced Release), a bioinformatics tool that identifies bacteriophage sequences within bacterial chromosomes. Further computational analyses were conducted to align bacteriophage proteins, genomes, and determine phylogenetic relationships amongst these viruses. This part of the project was carried out using the Clustal omega, MAFFT (Multiple Alignment using Fast Fourier Transform), and Interactive Tree of Life (iTOL) programs for sequence alignments and phylogenetic analyses. The laboratory activities were field tested in undergraduate directed research, and microbiology classes. The learning objectives were assessed by comparing the scores of pre and post-tests and grading final presentations. Post-tests were higher than pre-test scores at or below *p* = 0.002. The data suggest *in silico* phage hunting improves students’ ability to search databases, interpret phylogenetic trees, and use bioinformatics tools to examine genome structure. This activity allows instructors to integrate key bioinformatic concepts in their curriculums and gives students the opportunity to participate in a research-directed learning environment in the classroom.

## Introduction

There is a distinct need in life science education for educators to adapt their teaching strategies to best support student learning and prepare them for careers in science. Scientific education councils cite incorporating research into the undergraduate curriculum and emphasizing the interdisciplinary nature of biology as major national reform goals (National Research Council, 2009; AAAS, 2010). Research experiences are often interdisciplinary in nature and can teach students to think like scientists (AAAS, 2010; Ballen et al., 2017), greatly increase their chances of entrance into graduate school (Bangera and Brownell, 2014), and allow students to actively engage with current problems in biology. However, the process of obtaining a research experience while at college presents a variety of barriers to historically underrepresented and marginalized student populations (Stebleton and Soria, 2012). As a result, many students are left out of research experiences that can greatly enrich their understanding of biology, open doors for them professionally, and allow them to contribute their perspective in the larger scientific community (Bangera and Brownell, 2014). To make research experiences more accessible, many instructors opt to integrate research-based learning activities into their course curriculums. The activity we describe here is a low-cost research experience that can be implemented as a multi-week laboratory exercise in microbiology and other biology courses. This activity, which we have called *In Silico Phage Hunting*, utilizes bioinformatic tools to learn about bacteriophage genomes and viral proteins. We offer a perspective that this activity can help research experiences be more accessible by being integrated into appropriate elective biology courses, and engages students in inquiry-based learning, critical thinking, and scientific discovery which are all key components of the research process.

*In Silico Phage Hunting* is an adaptable teaching module that utilizes freely available bioinformatics software to examine the abundance of bacteriophages in bacterial genomes, the viral proteins encoded, and the phylogenetic relationships between phage proteins. Many bacterial genomes remain to be examined for the existence of bacteriophages. For example, only 500 sequenced genomes of bacteriophages that infect the genus *Escherichia* have been isolated compared to the 66,000 *Escherichia* bacterial genomes that are publicly available (Sazinas et al., 2017). Yet, bacteriophages contain a high proportion of novel genetic sequences and are likely to represent the largest reservoir of unexplored genes on earth (Hatfull, 2008). Given the existence of so many bacterial genomes in public databases and the limited knowledge of phages therein, locating phages can help bridge this gap in scientific understanding. *In Silico Phage Hunting* engages students in researching bacteriophage abundance and diversity while they learn about the genetic interplay between viruses and bacteria and develop important bioinformatics skills relevant for careers in STEM fields.

Bacteriophages have been used as model systems to incorporate scientific inquiry and research into undergraduate classrooms across the United States. The SEA-PHAGES (Science Education Alliance Phage Hunting Advancing Genomics and Evolutionary Science) program has successfully engaged thousands of students in authentic research through the isolation, characterization, and genome sequencing of bacteriophages from various bacterial species. Numerous laboratory exercises describing the use of phages to investigate diverse aspects of biology have been reported (Allen and Gyure, 2013; Hyman, 2014). Other initiatives include independent faculty members integrating phage research as multi-week laboratory exercises into their courses. Williamson et al. (2014) utilized a phage research experience in a molecular virology course to isolate novel bacteriophages and perform genetic analyses using computational tools. Recently, the Genome Solver Project utilized phage genomes to create hands-one bioinformatics activities and encourage educators to incorporate computational skills in their biology courses (Mathur et al., 2019). Despite these efforts, the number of phage-based laboratory activities incorporating genome searches, bioinformatics analyses, and phylogeny is still very limited in comparison to wet bench exercises.

Using computational methods to study bacteriophages is a promising area of biological research, as new insights can be uncovered about phage genomics and proteomics through bioinformatic analysis. Many studies have utilized the data present in public databases and bioinformatic analyses to research phage transcription (Guzina and Djordjevic, 2015), evolutionary classification (Lima-Mendez et al., 2008), and protein function (Carlton et al., 2005). Other authors advocate that undergraduate students can spearhead this research into bacteriophage abundance and diversity (Staub et al., 2017). In the Internet era, many more possibilities for scientific inquiry exist that previously were not accessible. The availability of microbial and viral genomes along with computational tools to assess these genomes gives teachers a unique opportunity to incorporate more inquiry-based and active learning exercises into their classrooms.

Bioinformatics skills are increasingly relevant to research in most areas of the life sciences. A recent nationwide faculty survey led to the development of a set of nine core competencies to guide the integration of bioinformatics in the life sciences curriculum (Wilson-Sayres et al., 2018). These competencies include but are not limited to: (1) understanding the role of data mining and computation in hypothesis-driven processes in the life sciences, (2) summarizing key computational concepts, (3) applying statistical concepts used in bioinformatics, (4) utilizing bioinformatics tools to analyze genomic information, and (5) knowing how to access genomic (Wilson-Sayres et al., 2018). A large number of publicly available bacterial genomes provides an excellent opportunity to teach about the abundance and evolutionary relationships amongst bacteriophages while incorporating five of the nine core bioinformatics competencies in microbiology courses.

*In Silico Phage Hunting* addresses five of these competencies by accessing genomic data from NCBI (National Center for Biotechnology Information), analyzing bacterial and phage genomes with bioinformatic tools, and facilitating group discussion throughout this process. Additionally, students prepare a final presentation or poster of their findings and complete worksheets along the way, acting as “check points” for their understanding of the concepts underlying the activity. This activity is also designed to promote inquiry in student groups by allowing enough time for students to discuss the concepts they are learning about and practicing the related skills. A sense of scientific discovery is also present in this activity, as students know they are locating phages in bacterial genomes and making genetic interpretations that have not yet been made.

## Lesson Overview

The *In Silico Phage Hunting* lesson described in this report was designed for upper level biology students. The activities can be conducted as part of the laboratory component of upper level biology classes or as independent projects in directed research courses. In traditional biology classes, the activities can be taught as multi-week laboratory exercises or separate classroom assignments. Prior to starting this activity, participants must have background knowledge of the following concepts: (1) principles of microbial and eukaryotic cell structure, (2) the central dogma of biology, (3) basic interpretation of phylogenetic trees, and (4) familiarity with sequence similarities searches and their application to biological research questions. It is recommended that students have completed at least one semester of genetics or cellular biology for effective participation in this project. If the students do not have substantial background in these areas, the instructor should incorporate brief lectures and discussions on common bioinformatics tools, genomes sequences, and any other themes considered necessary for the activity.

**FIGURE 1:**
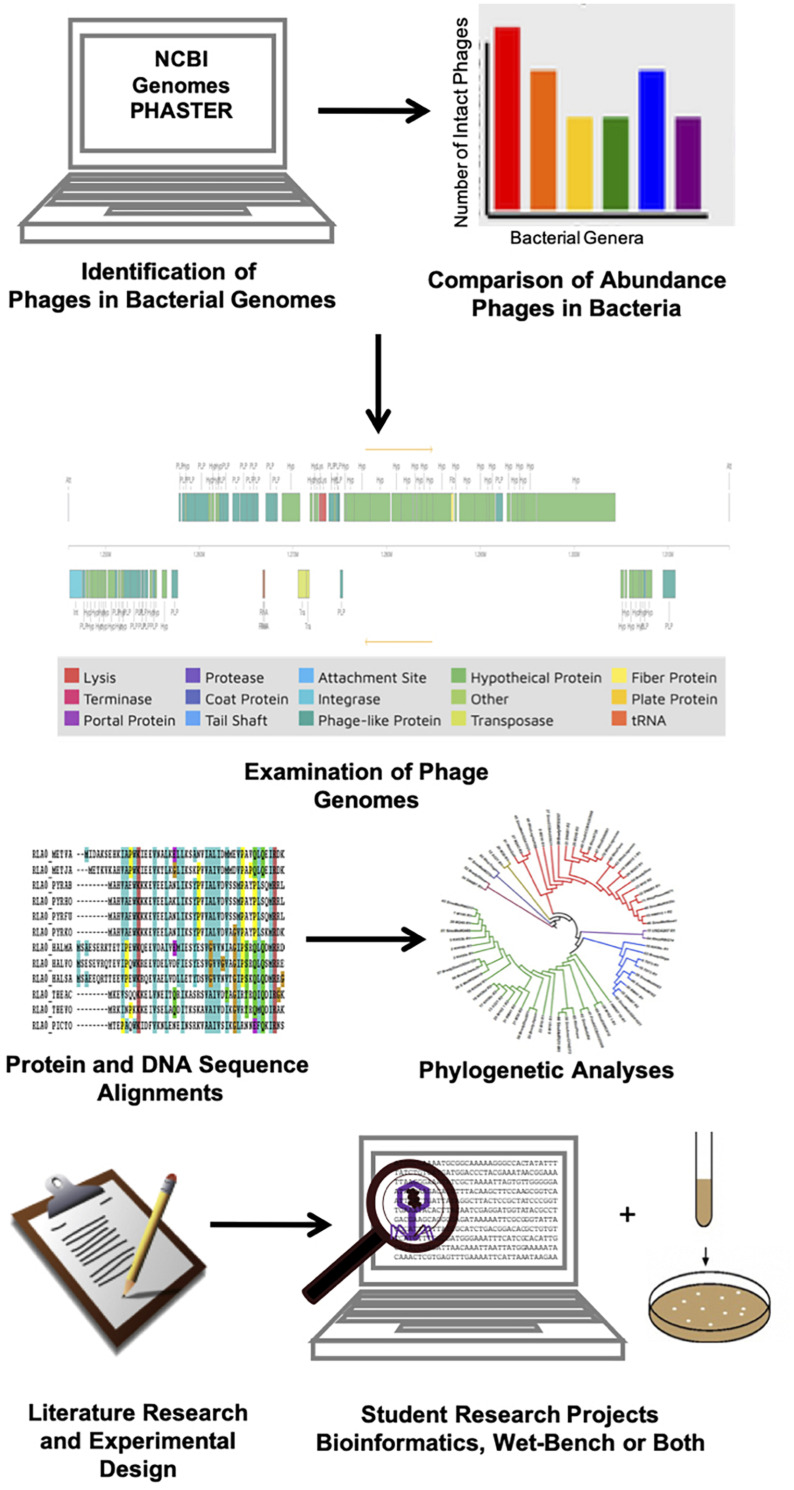
Workflow for *in silico* phage-hunting multi-week laboratory project conducted by microbiology students to investigate the presence of bacteriophages in the genomes of different bacteria.

The workflow for multi-week laboratory exercises and research projects is illustrated in Figure 1. During the first week, students have a pre-test followed by an assigned reading of the laboratory handout and a lecture on phage biology. Bioinformatics searches and phage genome exploration are covered in the second and third week of phage hunting activities. The fourth, fifth, and sixth weeks are used for experimental design, data retrieval, and analysis. Instructors have the choice of using additional weeks for wet-bench experiments or more computational analysis (Supplementary File S1). Independent research projects follow a timeline similar to the multi-week laboratory exercises, however, these students have additional weeks to expand their research questions and perform more detailed bioinformatics analyses (Supplementary File S1). Both the multi-week laboratory activity and the research project have a final assessment consisting of a poster or an oral presentation.

The *In Silico Phage Hunting* activities were designed to achieve the following learning objectives:

1. Describe the basic structure of a bacteriophage.
2. Explain the cycle of bacteriophage infection and replication.
3. Construct and interpret phylogenetic trees to investigate evolutionary relationships amongst phages.
4. Utilize bioinformatics tools to detect phages in bacterial genome sequences.
5. Retrieve bacteriophage genomes and protein sequences from public databases.
6. Formulate hypotheses regarding the abundance of bacteriophages in microbial genomes.

This research was deemed exempt status by the Hamline University IRB committee as defined by federal regulations (Final Common Rule, 45 CFR §46.104) under normal educational research. The study presented less than minimal risk associated with students’ participation and was conducted in an established educational setting using practices that were not likely to adversely impact student learning or assessment of the instructor providing the lesson. The data shown is anonymous and cannot be linked directly or indirectly to any of the participants in the study.

## Results and Discussion

The *In Silico* phage hunting activities were field-tested in two classes, Microbiology (AY 2015–2016), and Research in Biology (AY2017–2018). In lecture-based classes, these activities were conducted as part of multi-week investigative laboratory exercises in the laboratory component of the course. Students taking the Research in Biology course designed and completed independent projects using the *In Silico Phage Hunting* approach over a period of 6–10 weeks. Examples of laboratory and directed research projects are presented in Supplementary File S2.

Student learning after the completion of the *In Silico* phage hunting activities was evaluated using multiple assessment tools. For the lecture-based courses, we used the scores of pre and post-tests (Table 1 and Supplementary Files S3) to assess learning objectives (LOs) 1–5. The scores in the post-tests were significantly higher than the pre-tests with a *p*-value at or below 0.002. Learning gains were calculated for learning objectives 1–5. These analyses showed learning objectives 3–5 improved the most with learning gains equal to 0.50, 0.58, and 0.52, respectively. In contrast, LO1 and LO2 showed learning gains of 0.27 and 0.35. These results suggest the introductory lecture, and laboratory exercises completed in the initial portion of the phage-hunting activity improved students’ knowledge of bacteriophage biology, computational detection of phages, and interpretation of phylogenetic trees. The data from pre and post-tests indicate students showed the most improvement in the skills related to database searches and interpretation of phylogenetic trees (LOs 3–5).

**TABLE 1:** Summary of pre/post-test assessment data for *in silico* phage hunting laboratory activities field tested in microbiology courses.

**Course**	**Pre-test score (%)**	**Post-test score (%)**	***p*-value^*a*^**	**Normalized learning gains^*b*^**
Microbiology 2015 (*n* = 19)	60.1	71.7	0.00036	0.28
Microbiology 2016 (*n* = 13)	62.9	80.5	0.00179	0.47

*^a^A paired two tail t- test was used to evaluate the mean difference between pre and post-test scores. These scores were significant with p-values at or below 0.002.^b^Average normalized individual student learning gains (G) were calculated for the questions in the pre- and post-tests. G = [(post-test – pre-test)/(100 – pre-test)].*

Learning objectives 3–6 were designed to allow students to formulate hypotheses and employ publicly available data with computational tools to address these propositions. These activities were assessed by grading laboratory worksheets, and final research presentations (Supplementary Files S2, S8). Rubrics were developed to evaluate hypothesis statements, interpretation of bioinformatics data, and presentations (Supplementary File S5). Evaluation of laboratory worksheets showed students were able to formulate hypotheses and use publicly available genome data to test these propositions. Students were able to formulate hypotheses regarding the abundance of phages in *Escherichia coli*, and several genera of Nitrogen-fixing bacteria. In one of the courses, students carried out additional wet bench experiments to investigate induction of phages using diverse conditions such as temperature shifts, exposure to UV light, and chemicals. When students did *In Silico Phage Hunting* as a summer or semester long research project, they often conducted phage induction and isolation experiments as part of their projects (Supplementary File S2). The laboratory worksheets (Supplementary File S8) and presentations showed most students met expectations regarding the formulation of hypotheses, retrieval of data from public repositories and interpretation of phylogenetic trees. Students’ verbal feedback, overall attitude, and level of engagement with the *In Silico Phage Hunting* activities were very positive. These observations suggest they had an appreciation for bioinformatics and its applications to biology.

*In Silico Phage Hunting* provides multiple opportunities for students to be exposed to and practice core bioinformatics competencies (Supplementary File S6). Formulating hypotheses about the abundance of phages in microbial genomes highlights the role of computation and data mining in addressing questions in the life sciences; this is one of the most important bioinformatics competencies. In addition, during the phage hunting activities, students used multiple databases and evaluated statistical values to assess the accuracy of bioinformatics predictions. These skills support the development of various bioinformatics competencies including the use of computational tools to examine biological problems and applying statistical concepts in bioinformatics.

Students also practice appropriate retrieval and organization of large data sets. These competencies are essential when locating and sorting through different types of biological data to construct sequence alignments and phylogenetic trees.

*In Silico Phage Hunting* was designed as a series of multi-week laboratory exercises, therefore, the activity has many fundamentally “hands-on” active learning components. For example, students were engaged in downloading data from public databases, employing bioinformatics web tools to analyze bacterial and viral genome sequences, and using the information gathered to discuss the biological relevance and implications of their findings. Many parts of this activity involve students working in teams of 3–4 people. This strategy leads to group discussions focused on the value of the information gathered, and whether the results obtained support or refute the hypothesis posed. These activities are all consistent with the definition of activity learning which states “anything that involves students in doing things and thinking about the things they are doing” (Bornwell, 1991). Group work also encourages effective communication, and higher-order thinking tasks such as critical data analysis, evaluation and synthesis of information.

By using freely available Internet software in research, these activities provide students of diverse backgrounds and academic abilities with an opportunity to learn how to use bioinformatic tools to test hypotheses. Laboratory projects involving data mining and bioinformatics prepare students to participate in summer or course-based research experiences given that addressing modern scientific questions in biology often involves working with large data sets as well as retrieving information from databases. The activities carried out as part of these projects make use of multiple approaches to teach about bacteriophage structure, function, and diversity. Students formulate hypotheses, perform database searches, and explore different ways to analyze and present data. These exercises provide students of diverse learning styles with an opportunity to engage with topics being taught and make contributions to their team.

The phage hunting activities can be easily modified and carried out with any bacterial species, offering research opportunities for students in the classroom that otherwise would be difficult to access. *In Silico* Phage Hunting also provides prospects to discover and investigate novel phages in bacterial genomes. An extension of these activities could include searching and cataloging the abundance of RNA phages in bacterial chromosomes. Once a complete DNA or RNA phage genome is predicted by PHASTER, investigators can isolate the virus by induction of the lytic cycle or cloning. This type of research can contribute to enhancing our understanding of phage biology and viral diversity.

Phage genome analyses can be modified to incorporate other viral bioinformatic tools such as: the MVP (Microbe-Vs.-Phage) database, VIRFAM (Viral Protein Families), and Phage Signature Genes, PhiSiGns (Supplementary File S7). *In Silico Phage Hunting* is suitable as a research project or as a laboratory activity for upper-level courses such as virology, microbiology, evolution, and molecular biology. Alternatively, instructors can use the phage hunting activities as individual or stand-alone modules to create assignments and supplement class content. Many of the bioinformatics activities are easy to follow and can be used as classroom or one-time laboratory exercises. These activities can also be used together with commercially available bacteriophage induction and plaque demonstration kits.

## Perspective

The equitable access of research experiences is of concern in academia. We know students benefit greatly from these experiences. Students report that they have a greater sense of pride in their work (Hekmat-Scafe et al., 2017), learn critical thinking skills by virtue of engaging in the research process, and gain a greater sense of awareness about what scientific research is like. Research experiences are also one of the crucial keys for entrance into graduate programs (Bangera and Brownell, 2014). Given the high importance of research experiences in relation to student learning, appreciation of biology, and gateways to academic and professional opportunities, these experiences should be designed to be more accessible to a wider range of students. Scientific educators occupy a critical space by being able to directly implement research experiences in their classrooms. As individual teachers, we must consider what we can do to create an equitable learning environment for all learners. Teachers have a direct relationship with students’ education, and it is this education that can open doors for students into future careers as educators, researchers, doctors, and scientists. Research experiences can open these doors for students and empower them as learners. Teachers can implement a tangible change in their classrooms by incorporating research experiences into their curriculums.

*In Silico Phage Hunting* is a classroom activity that engages students in important aspects of the research process: hypothesis testing, data mining, interpretation of results, and sharing of findings. It is an adaptable teaching module that incorporates research into laboratories and classrooms with little cost to instructors. A barrier to incorporating research experiences into classrooms is often an issue of lack of resources and funding for departments. The activity we described in this paper does not require advanced equipment or massive amounts of funding to implement and was field-tested at a small liberal arts college showing learning gains made by students. Biological databases offer a new approach to incorporating research experiences into classrooms that would otherwise have been difficult to achieve.

By developing a teaching module that utilizes research into bacteriophage abundance and diversity, we aim to confront the barriers present in academic research. The wealth of biological data that is freely available in databases such as NCBI (National Center for Biotechnology Information) presents a unique opportunity that was not available for past educators to incorporate research into the classroom. Software tools available on the Internet present new modes of inquiry into this data, offering new interpretations and insights into bacteriophage abundance and diversity. The core of this activity is the use of freely available data and tools on the Internet to design research activities in the classroom, and we encourage other educators to get creative with this accessible information.

## Data Availability Statement

The raw data supporting the conclusions of this article will be made available by the authors, without undue reservation, to any qualified researcher.

## Author Contributions

MM and BM-V contributed to different sections of this work. BM-V devised the study, created the Supplementary Material, and authored the sections “Results and Discussion” and the “Lesson Overview.” MM performed one the research projects, authored the sections “Introduction” and “Perspective”, and managed the references. Both authors contributed to the article and approved the submitted version.

## Conflict of Interest

The authors declare that the research was conducted in the absence of any commercial or financial relationships that could be construed as a potential conflict of interest.
