# Supplementary material for: In silico Phage Hunting: Bioinformatics Exercises to Identify and Explore Bacteriophage Genomes
Source: Front Microbiol. 2020 Sep 17;11:577634. doi: 10.3389/fmicb.2020.577634 (PMC7533560; doi:10.3389/fmicb.2020.577634)
Supplement: Supplementary file 1 [file Data_Sheet_1.PDF]

Supporting File S1: Timeline for Phage Hunting Activities

Week	Activity_Multiweek Lab	Description	Learning Objectives
1	• Pre-test<br>• Assigned reading of laboratory handout<br>• Bacteriophage Lecture<br>• *Worksheet I:* Bacteriophages in microbial genomes	Students listen to a lecture to learn about bacteriophage structure and function.<br><br>Students work on a worksheet to think about the abundance and relevance of phages.	LO1, LO2
2	• Exploring and Retrieving Information from the NCBI Genome Database<br>• PHASTER Tutorial Exploring the PHASTER Tool<br>• *Worksheet II:* Database Exploration, Data Retrieval, and PHASTER Tool	Work on browsing the NCBI genome database and complete the data retrieval and database exploration worksheet.<br><br>Watch the PHASTER tutorial and explore the PHASTER tool.	LO4, LO5
3	• DNA and Protein Sequence Alignments<br>• Constructing and Presenting Phylogenetic trees<br>• *Worksheet III:* Sequence Alignments and Phylogenetic trees	Practice doing DNA and protein sequence alignments with data provided by the instructor.<br><br>Generate phylogenetic trees and use iTOL to visualize and edit tree files.	LO3, LO4
4	*In Silico* Phage Hunting: Experimental Design<br><br>*Worksheet IV:* Formulating Hypotheses and Designing an Experimental Plan	Formulate hypotheses that can be addressed using the computational tools studied in weeks 1-3.	LO6
5 &6	*In Silico* Phage Hunting: Data Collection and Analysis<br>• PHASTER analysis in bacterial genomes of interest<br>• Retrieval of phage genomes, and protein of interest<br>• DNA and protein sequence alignments<br>• Construction of phylogenetic trees<br>• Post Test	Students work in groups to collect and analyze data. They have discussions about the results and how they support or refute the hypotheses posed. This part of the activity can last one or two weeks due to students' levels of ability, feasibility of the project and technical challenges.	LO3, LO4, LO5
7	*In Silico* Phage Hunting: Final Presentations	Final oral presentations to share the findings of "in silico" phage hunting projects.	LO6

Supporting File S1: Timeline for Phage Hunting Activities

Week	Activity_Directed Research Projects	Description	Learning Objectives
1	• Assigned reading of laboratory handout and research paper<br>• *Worksheet I:* Bacteriophages in microbial genomes	Students listen to a lecture to learn about bacteriophage structure and function.<br><br>Students work on a worksheet to think about the abundance and relevance of phages.	LO1, LO2
2	• Exploring and Retrieving Information from the NCBI Genome Database<br>• PHASTER Tutorial Exploring the PHASTER Tool<br>• *Worksheet II:* Database Exploration, Data Retrieval, and PHASTER Tool	Work on browsing the NCBI genome database and complete the data retrieval and database exploration worksheet.<br><br>Watch the PHASTER tutorial and explore the PHASTER tool.	LO4, LO5
3	• DNA and Protein Sequence Alignments<br>• Constructing and Presenting Phylogenetic trees<br>• *Worksheet III:* Sequence Alignments and Phylogenetic trees	Practice doing DNA and protein sequence alignments with data provided by the instructor.<br><br>Generate phylogenetic trees and use iTOL to visualize and edit tree files.	LO3, LO4
4	*In Silico* Phage Hunting: Experimental Design<br>*Worksheet IV:* Formulating Hypotheses and Designing an Experimental Plan	Formulate hypotheses that can be addressed using the computational tools studied in weeks 1-3.	LO6
5 -9	*In Silico* Phage Hunting: Data Collection and Analysis<br>• PHASTER analysis in bacterial genomes of interest<br>• Retrieval of phage genomes, and protein of interest<br>• DNA and protein sequence alignments<br>• Construction of phylogenetic trees<br>• Bench Experiments: Prophage Induction, PCR, Phages isolation, etc.	Students work in groups to collect and analyze data. They have discussions about the results and how they support or refute the hypotheses posed. This part of the activity can last one or two weeks due to students' levels of ability, feasibility of the project and technical challenges.	LO3, LO4, LO5
10	*In Silico* Phage Hunting: Final Presentations or Posters	Final oral presentations or posters to share the findings of "in silico" phage hunting projects.	LO6
