# Supplementary material for: In silico Phage Hunting: Bioinformatics Exercises to Identify and Explore Bacteriophage Genomes
Source: Front Microbiol. 2020 Sep 17;11:577634. doi: 10.3389/fmicb.2020.577634 (PMC7533560; doi:10.3389/fmicb.2020.577634)
Supplement: Supplementary file 2 [file Data_Sheet_2.PDF]

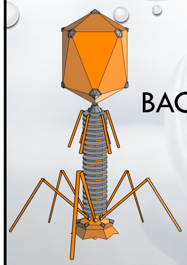

BACTERIOPHAGES IN FOOD BORNE PATHOGENS

1

BACKGROUND

- Food borne illness affected 600 million people and caused 420,000 deaths globally in 2010 (world health organization [WHO], 2015)
- Bacteria which are members of the enterobacteriaceae family such as *E.coli* and *Salmonella* strains are commonly associated with foodborne illness worldwide (Thomas et al., 2015).
- Phages are known to carry bacterial toxins and antibiotic resistance genes in their genomes (Curiosa et al., 1999; Bielaszewska et al., 2012)

2

RESEARCH QUESTIONS

- Are bacteriophages more abundant in bacterial strains associated with foodborne illness compared to commensal strains?
- Are bacteriophages that infect *Salmonella* evolutionary related to phages that infect *Escherichia coli*?

3

HYPOTHESES

- Pathogenic strains of *Escherichia coli* and *Salmonella* contain more bacteriophages than commensal strains of the same organism.
- The bacteriophages that infect *Escherichia coli* share a close evolutionary relationship with the phages that infect *Salmonella* strains.

4

METHODS

- Retrieve *E.coli* and *Salmonella* Genomes
  - Commensal and Pathogenic Strains
- PHASTER Analysis
  - Download and save genome and protein files
- Genome and Protein Sequence Alignments

5

Results: Intact phages In Diverse *E.coli* strains

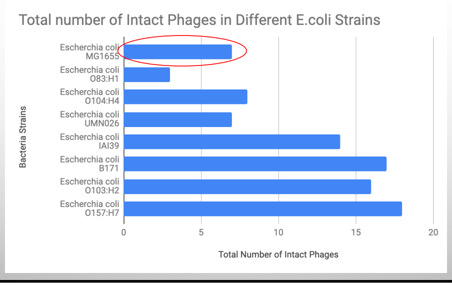

6

Results: Intact Phages in *Salmonella* Strains

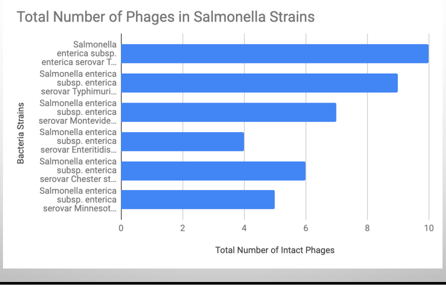

7

Prophages in the genome of *Escherichia coli* 0157:H7 and *Salmonella enterica subsp. enterica serovar Typhi* str. CT18

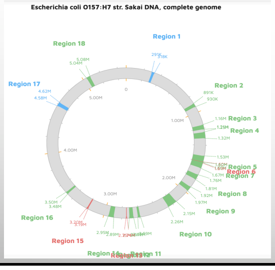

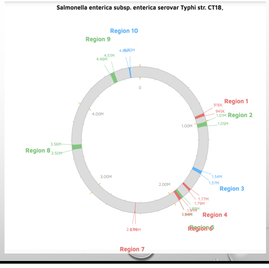

8

Genome structure of *E.coli* O157:H7 Prophage 4

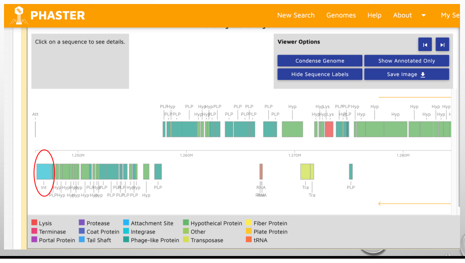

9

Virulence Associated proteins in *Escherichia coli* 0157:H7 Phages

PHASTER — New Search | Genomes | Help | About | My Se

39	complement(2920734..2921267)	PHAGE_Entero_933W_NC_000924: endolysin; ECs_2968; phage(gi9632511)	2.02e-128	Show
40	complement(2921272..2921487)	PHAGE_Shigel_Stx_NC_029120: holin lysis protein S; ECs_2969; phage(gi985761286)	8.61e-46	Show
41	complement(2921565..2921810)	PHAGE_Escher_P13374_NC_018846: hypothetical protein; ECs_2970; phage(gi410491644)	1.77e-50	Show
42	complement(2921851..2922030)	PHAGE_Stx2_vB_EcoP_24B_NC_027984: hypothetical protein; ECs_2971; phage(gi937456280)	3.06e-38	Show
43	complement(2922168..2924114)	PHAGE_Escher_phage_NC_004913: hypothetical protein; ECs_2972; phage(gi32170979)	0.0	Show
44	complement(2924625..2924894)	PHAGE_Entero_YYZ_2008_NC_011356: Shiga toxin 1 subunit B; ECs_2973; phage(gi209427764)	1.23e-60	Show
45	complement(2924904..2925851)	PHAGE_Entero_YYZ_2008_NC_011356: Shiga toxin 1 subunit A; ECs_2974; phage(gi209427763)	0.0	Show
46	complement(2926358..2926840)	PHAGE_Escher_phage_NC_004913: Q protein; ECs_2975; phage(gi32170974)	2.47e-114	Show

10

Virulence Associated proteins in *Salmonella* Phages

PHASTER — New Search | Genomes | Help | About | My Searches

6	1782934..1783146	putative cold shock protein; -	0.0	Show
7	1783564..1784085	putative lipoprotein; -	0.0	Show
8	1784276..1784515	PHAGE_Entero_HK225_NC_019717: DNA damage-inducible protein; -; phage(gi428782403)	7e-35	Show
9	complement(1784727..1784933)	PHAGE_Entero_Sf6_NC_005344: putative transposase OrfB; -; phage(gi41057343)	6e-05	Show
10	1785388..1786197	PHAGE_Entero_cdtI_NC_009514: Cytolethal distending toxin B subunit; -; phage(gi148609410)	1e-63	Show
11	1786270..1786647	PHAGE_Pseudo_Lu11_NC_017972: putative tail assembly protein; -; phage(gi388684690)	9e-16	Show
12	complement(1786795..1787337)	PHAGE_Erwini_vB_EamP_S6_NC_019514: endolysin; -; phage(gi422935930)	3e-63	Show
13	complement(1787529..1788257)	putative pertussis-like toxin subunit; -	0.0	Show
14	complement(1788274..1788687)	putative pertussis-like toxin subunit; -	0.0	Show
15	complement(1789728..1790852)	putative bacteriophage protein; -	0.0	Show
16	1791034..1791046	attR	0.0	Show

11

Phylogenetic Tree of *E.coli* and *Salmonella* Integrases

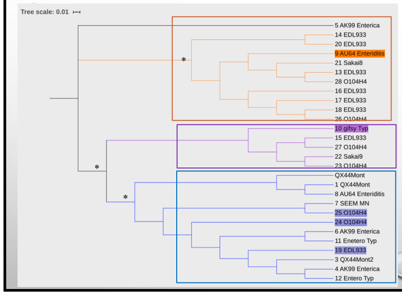

12

DISCUSSION

Hypothesis I: Pathogenic strains of Escherichia coli and Salmonella contain more bacteriophages than commensal strains of the same organism.

- Amongst the strains studied, pathogenic *E. coli* had more prophage sequences than the non-pathogenic strain *E.coli* K-12 MG1655
- All Salmonella strains analyzed had been linked to food borne illness or other pathologies, we did not have a non-pathogenic control
- Proteins associated with virulence were identified in the genomes of both *E.coli* and *Salmonella* phages
- The higher abundance of prophages in pathogenic *E.coli* strains and the presence of Shiga Toxin proteins in these viruses partially supports the first hypothesis of the study.

13

DISCUSSION

- *Hypothesis 2: The bacteriophages that infect Escherichia coli share a close evolutionary relationship with the phages that infect Salmonella strains.*

- The phylogenetic tree showed tree clear groups, two made of *E.coli* phage integrases and another group of mostly *Salmonella* integrases.
- Integrases from some phages that infect *E.coli* strain O104:H4 were closely related to integrases from *Salmonella* phages
- The phylogenetic tree build with bacteriophage integrases suggests that *E. coli* phages were more closely related to themselves than to phages that infect *Salmonella*.
- The phylogenetic tree showed a distant ancestor for integrases found in phages that infect E.coli and *Salmonella*; this result partially supports hypothesis 2.

14

LIMITATIONS

- Only a limited number of integrases from each prophage were analyzed
- Other phage proteins could have been used to assess evolutionary relationships
- Lack of non-pathogenic strains for *Salmonella* comparisons

15

FUTURE WORK

- Expand the study to other foodborne pathogens
- Include gram positive and gram negative bacteria
- Search phage genomes for other toxins and antibiotic resistance genes

16

Diversity of Bacteriophages in the Genomes of Nitrogen-Fixing Bacteria

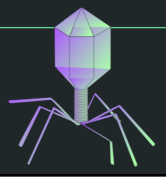

1

Bacteriophage: A Virus that Infects Bacteria

- Most abundant biological entity on earth
- Genomes range from encoding only 4 genes to 100+ genes
- Numerous biotechnical applications
  - Drug delivery systems *(Kovacs et al., 2007)*
  - Agricultural control *(Frampton, Pitman, & Fineran, 2012)*
  - Phage therapy *(Mendes et al., 2014)*

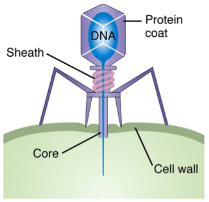

2

Phage Replication

- Takes place in the host bacterium
  - Lytic or lysogenic
- Prophage: segment of phage DNA integrated into a bacterial chromosome
  - Able to produce phages when activated
  - Contribute to horizontal gene transfer *(Santamaria et al., 2014)*

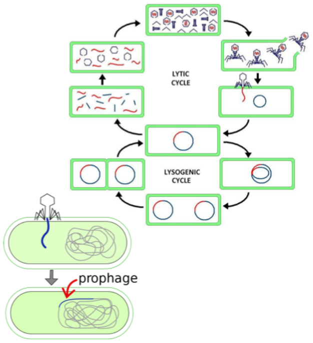

3

Phages and Nitrogen-Fixing Bacteria

- N-fixing bacteria found in zone of soil called the rhizosphere
  - Diverse community of microbes that influence soil ecology
- Form symbiotic relationship with plant roots
  - Fix nitrogen in nodules
    - Key role in promoting plant growth
- Rhizobium phages influence bacterial evolution and soil ecology

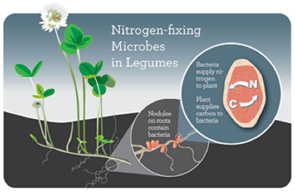

4

Significance of Study

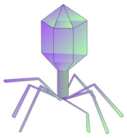

- **Phages in the rhizosphere have important agricultural applications**
  - Biocontrol of plant pathogens *(Frampton, Pitman, & Fineran, 2012)*
  - Modify rate of N-fixation *(Basit et al, 1992)*
  - Affect the survival and select for specific nitrogen-fixing bacteria *(Brussow, Canchaya, & Hardt, 2004)*
- **Gap in research on bacteriophage genomic studies**
  - Phages could represent the largest reservoir of unexplored genes *(Hatfull, 2008)*
  - Need for classification of phages, understanding their life cycle, and making evolutionary connections to bacterial hosts

5

Research Questions

➔ How abundant are bacteriophages in the genomes of Nitrogen-fixing bacteria?

➔ How evolutionary related are the bacteriophages found in rhizobia?

➔ Can we design a PCR-based assay to detect rhizophages in soils?

6

Methods

7

Bioinformatic Analyses

Phylogenetic Tree Construction

Development of a Primer Set

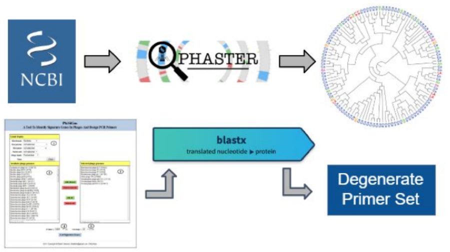

8

Genera	Accession Numbers	Number of Prophage Regions	Number of Intact Prophage Regions
	chromosome: NC_003047.1/AL591688.1	1	0
	plasmid pSymA: NC_003037.1/AE006469.1	1	0
Sinorhizobium meliloti 1020	plasmid pSymB: NC_003078.1/AL591985.1	1	0
	chromosome: NC_009636.1/CP000738.1	3	1
	plasmid pSMED01: NC_009620.1/CP000739.1	0	0
	plasmid pSMED02: NC_009621.1/CP000740.1	0	0
Sinorhizobium medicae WSM419	plasmid pSMED03: NC_009622.1/CP000741.1	0	0
	chromosome: NC_012587.1/CP001389.1	0	0
	plasmid pNGR234a: NC_000914.2/U00090.2	0	0
Sinorhizobium fredii NGR234	plasmid pNGR234b: NC_012586.1/CP000874.1	0	0
	chromosome: NC_016812.1/HE616890.1	3	0
	plasmid pSfHH103a: NC_016813.1/HE616891.1	0	0
	plasmid pSfHH103b: NC_016836.1/HE616892.1	0	0
	plasmid pSfHH103c: NC_016814.1/HE616893.1	1	0
Sinorhizobium fredii HH103	plasmid pSfHH103e: NC_016815.1/HE616899.1	0	0
	chromosome: NZ_CP016450.1/CP016450.1	1	1
	plasmid pBSY16_1: NZ_CP016452.1/CP016452.1	3	0
Sinorhizobium sp. RAC02	plasmid pBSY16_2: NZ_CP016451.1/CP016451.1	0	0
	chromosome: NZ_CP013051.1/CP013051.1	1	0
	plasmid A: NZ_CP013052.1/CP013052.1	2	0
	plasmid B: NZ_CP013053.1/CP013053.1	0	0
Sinorhizobium americanum CCGM7	plasmid C: NZ_CP013054.1/CP013054.1	1	0
	chromosome: NZ_CP023063.1/CP023063.1	1	0

- Obtained prophage sequences from *Sinorhizobium*, *Rhizobium*, *Bradyrhizobium*, and *Mesorhizobium* species
  - Only analyzed intact (complete) prophage regions

9

PHASTER: Finding Phages in Bacterial Genomes

- In this sample, we can see the whole genome of *Sinorhizobium meliloti AK83*
- This genome contains 1 intact phage (Region 3) and several other incomplete phage regions

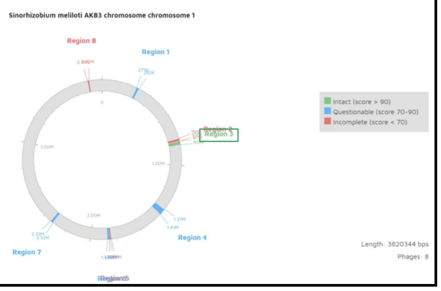

10

PCR Experiments with a Degenerate Primer Set

Positive Controls

Experimental Soil Samples

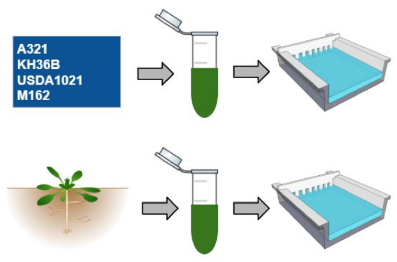

11

Results & Discussion

How abundant are bacteriophages in the genomes of Nitrogen-fixing bacteria?

12

Prophage Regions in Nitrogen-Fixing Bacteria

- Overall, 498 bacterial chromosome/plasmid sequences were obtained
  - 113 contained intact prophage regions

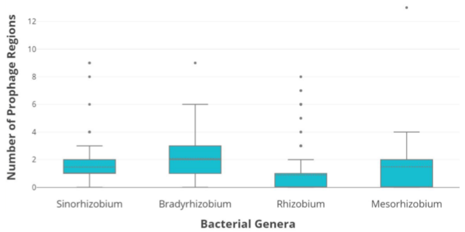

13

Percent of Intact Prophage Regions per Genera

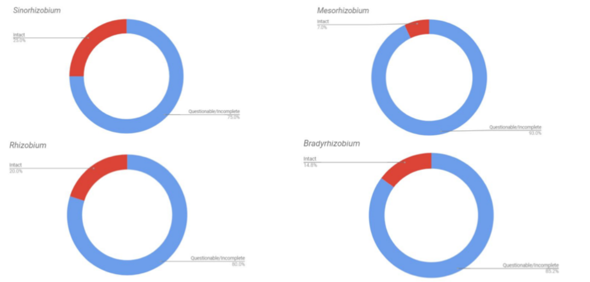

14

How Abundant are Phages in Rhizobia?

- 22% of the rhizobium genomes analyzed contained intact prophage regions
  - Only 500 phages found from over 66,000 genomes of *Escherichia* *(Sazinas et al., 2017)*
    - Less than 1% of *Escherichia* genomes contain phages
  - Rhizobium phages likely very abundant
- Other studies sampled fully annotated phage genomes to assess abundance *(Decewicz et al., 2017)*
  - This study analyzed intact prophage regions identified by PHASTER

15

Results & Discussion

How evolutionary related are the bacteriophages found in rhizobia?

16

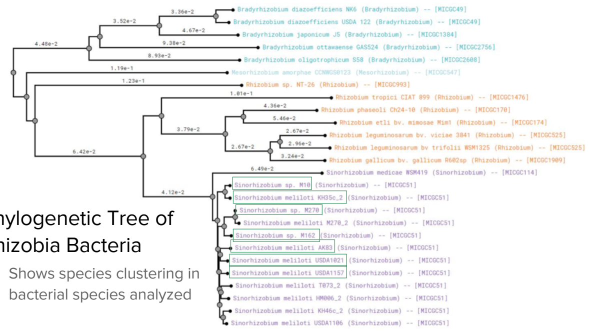

Phylogenetic Tree of Rhizobia Bacteria

- Shows species clustering in bacterial species analyzed

17

Phylogenetic Tree of Phages

- Shows evolutionary relationships between phages in different rhizobium strains
  - *Rhizobium, Sinorhizobium, Mesorhizobium,* and *Bradyrhizobium*
- What we expected:
  - Phages that infect the same type of bacterial host will cluster together

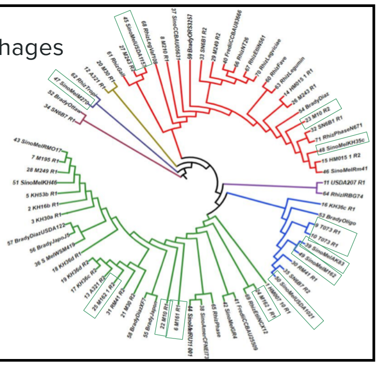

18

How Genetically Diverse are Phages in Rhizobia?

- Phylogenetic trees showed more genetically distinct groups of phages than there are rhizobia groupings
  - Could indicate that rhizobia phages are even more diverse than their bacterial hosts
- Most data came from *Sinorhizobium meliloti,* which could skew results
- Many phages contain novel genes, with little similarity at the genomic level *(Adriaenssens & Cowan, 2014)*
  - Presence of proteins with unknown function (hypothetical proteins)
    - Need for phage protein annotation to fully assess diversity

19

Results & Discussion

Can we design a PCR-based assay to detect for rhizophages in soils?

20

Designing a Degenerate Primer Set to Amplify a Phage Gene

PhiSiGns
A Tool To Identify Signature Genes In Phages And Design PCR Primers

List of potential primer pairs for selected SiG_1

There are 2 predicted primer pairs in the list below.

Pair	Forward Primer	Length	Degeneracy	Reverse Primer	Length	Degeneracy	Product size	T_m mismatch [?]	Compl. [?]	
1	GAYGGCSSGMTTRYCC	16	64	TGGAWMWKMGGCCSGC	16	64	406	0.05	Warning	Show Details
2	GAYGGCSSGMTTRYCC	16	64	STGGAWMWKMGGCCSG	16	128	407	0.02	Warning	Show Details

Primer Parameters	Min	Max
Primer Length (nt) [?]	16	28
GC content (%) [?]	30	80
Basic Melting Temperature (°C) [?]	30	80
Salt-Adjsuted Melting Temperature (°C) [?]	30	80
Nearest-Neighbor Melting Temperature (°C) [?]	30	80
Minimum Delta G (kcal/mol) [?]	-20	
Product Length (nt) [?]	400	2000
Primer Degeneracy [?]		128
3' GC Clamp [?]		no
Maximum 3' stability [?]		no
Complementarity [?]		no

Methyltransferase gene

List of genes in selected SiG_1

NCBI protein ID	Protein function	Length (nt)	Phage	Phage family	Start position	End position
NP_542323.1	Phage protein	564	Sinorhizobium phage PBC5	Unclassified	47836	48399
YP_002117654.1	Phage protein	642	Rhizobium phage 16-3	Siphoviridae	51381	52022

21

Amplification of a Conserved Phage Gene

- Applied findings from bioinformatic analysis to develop a degenerate primer set
  - A methyltransferase was identified as a conserved gene
- Used *Sinorhizobium meliloti* strains A321, KH36B, USDA1021, and M162
  - M162 showed no product

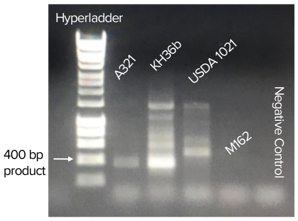

22

Experimental Soil Samples

- Used leguminous plants to detect for the presence of phages
  - *Sinorhizobium meliloti A321* included as positive control
- Faint banding in Honey Locust and Red Bud lanes
  - Need to optimize PCR procedure to be sure

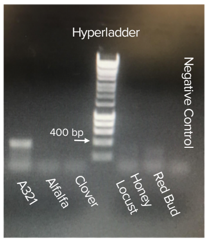

23

How Can PCR Detect for the Presence of Phages?

- Identifying signature genes
  - Difficult--phages have no universal genetic marker
- Other studies have successfully used PhiSiGns primer design for *gokushovirus* *(Hopkins et al)*
- PCR-based identification of signature genes is most used, but not adequate enough
  - Majority of viral sequences don't have a database counterpart *(Adriaenssens & Cowan, 2014)*
- Need to identify more signature genes to develop different primer sets

24

Summary of Findings

25

➔ How abundant are bacteriophages in the genomes of Nitrogen-fixing bacteria?

- Retrieved publicly available rhizobia genomes
  - Analyzed them using PHASTER to detect for prophage regions
- Found 498 bacterial genomes, with 113 intact prophage regions
  - Ranged from containing only 1 intact prophage region, to 13 regions
- Determined that rhizobial phages are very abundant

PHASTER

26

➔ How evolutionary related are the bacteriophages found in rhizobia?

- Created two phylogenetic trees to compare rhizobia and their phages
- Phage phylogenetic tree formed 7 distinct groups, while rhizobia tree had 4 distinct groups
- Results indicate that rhizobium phages are very diverse, but more testing needed to be more precise
  - Creating a merged tree showing coevolution of phages and rhizobia

27

➔ Can we design a PCR-based assay to detect rhizophages in soils?

- Designed a degenerate primer set using PhiSiGns
- Used *Sinorhizobium meliloti* strains found to contain a signature gene (methyltransferase)
  - Amplification in all but M162 strain
- Soil samples showed no concrete amplification
  - Optimize the PCR procedure or look for more signature genes

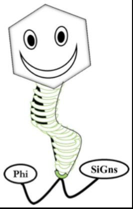

28

Limitations of Study

- Use of degenerate primers could have lead to non-specific amplification
  - Could explain why gel results appeared ‘streaky’
- Bacteria are constantly evolving
  - Need for updating findings to keep up with newly sequenced genomes
- Could use better bioinformatics software to analyze data, requires more advanced computer systems

29

Future Directions for Research

- Expand the bioinformatics analyses to include more genera of bacteria found in soil
- Develop additional primer sets which contain phage proteins for detection of phages in soil samples
- Perform and optimize the polymerase chain reaction for bacterial soil samples
- Construct phylogenetic trees for specific phage proteins

30

Works Cited

Hatfull G. F. (2008). Bacteriophage genomics. *Current opinion in microbiology*, *11*(5), 447-53.

Frampton, R. A., Pitman, A. R., & Fineran, P. C. Advances in Bacteriophage-Mediated Control of Plant Pathogens. International Journal of Microbiology. doi:10.1155/2012/326452

Basit H. A., Angle J. S., Salem S., Gewaily E. M. (1992). Phage coating of soybean seed reduces nodulation by indigenous soil bradyrhizobia. *Can. J. Microbiol.* 38 1264–1269. 10.1139/m92-208

Decewicz, P., Radlinska, M., & Dziewit, L. (2017). Characterization of Sinorhizobium sp. LM21 Prophages and Virus-Encoded DNA Methyltransferases in the Light of Comparative Genomic Analyses of the Sinorhizobial Virome. *MDPI Viruses, 9(161)*. doi:10.3390/v9070161

Adriaenssens, E. M. & Cowan, D. A. (2014). Using Signature Genes as Tools To Assess Environmental Viral Ecology and Diversity. *Applied and Environmental Microbiology, 80(15)*, 4470-4480.

Brussow, H., Canchaya, C., & Hardt, W. D. (2004). Phages and the evolution of bacterial pathogens: from genomic rearrangements to lysogenic conversion. *Microbiol. Mol. Biol. Rev.* 68:560–602.doi:10.1128/MMBR.68.3.560-602.2004.

Kovacs, E. W., Hooker, J. M., Romanini, D. W., Holder, P. G., Berry, K. E., & Francis, M. B. (2007). Dual-Surface-Modified Bacteriophage MS2 as an Ideal Scaffold for a Viral Capsid-Based Drug Delivery System. *Bioconjugate Chemistry, 18(4),* 1140-1147. doi:10.1021/bc070006e

Mendes, J. J., Leandro, C., Mottola, C., Barbosa, R., Silva, F. A., Oliveira, M., Vilela, C. L. … & Garcia, M. (2014). In vitro design of a novel lytic bacteriophage cocktail with therapeutic potential against organisms causing diabetic foot infections. Journal of Medical Microbiology, 63, 1055-1065. doi:10.1099/jmm.0.071753-0

Bohannan, B.J.M. & Lenski, R.E. (2002). Linking genetic change to community evolution: insights from studies of bacteria and bacteriophage. *Ecology Letters, 3(4)*. doi:10.1046/j.1461-0248.2000.00161.x

31

Induction of *Sinorhizobium* Prophages by Ultraviolet Light & Mitomycin

1

Background

- What is a bacteriophage?
  - Virus that infects bacteria
  - Lytic and lysogenic phases
- Present at ~10^9 virions per 1g in soil

2

Purpose

- Not a large amount of information about phages that infect plant growth promoting bacteria like *Sinorhizobium;* only seven rhizophage genomes have been sequenced as of 2014
- If induced and isolated than further studies can be done to investigate:
  - the role of phages in the transfer of genes involved in plant microbe interactions
  - presence of genes linked to survival in the environment
  - presence of new genes potentially involved in symbiosis

3

Method: Bioinformatics

MaGe was used to export Fasta versions of 51 *Sinorhizobium* genomes to PHAST

→

The PHAST program then searched the genomes for prophages, categorizing them as “intact,” “incomplete,” and “questionable”

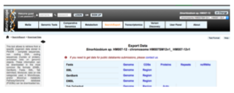

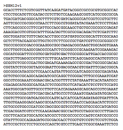

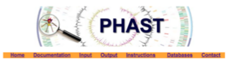

4

Method: UV Induction

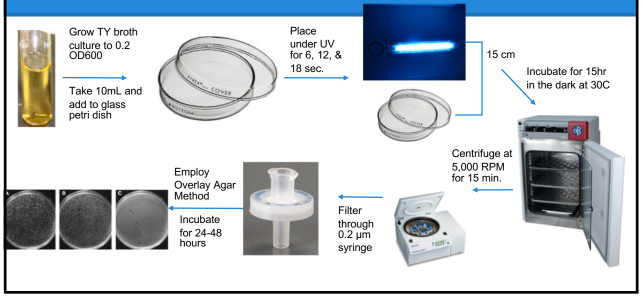

5

Method: Mitomycin Induction

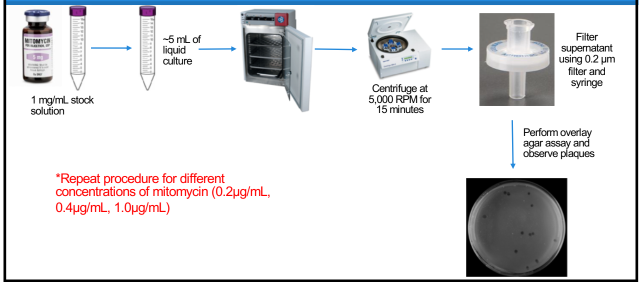

*Repeat procedure for different concentrations of mitomycin (0.2µg/mL, 0.4µg/mL, 1.0µg/mL)

6

Results: Bioinformatics

Total : 6 prophage regions have been identified, of which 2 regions are intact, 1 regions are incomplete, 3 regions are questionable

REGION	REGION_LENGTH	COMPLETENESS	SCORE	#CDS	REGION_POSITION	POSSIBLE PHAGE
1	8.1Kb	questionable	90	15	596048-604212	PHAGE_Stx2_converting_1717_NC_011357, ...
2	14.7Kb	questionable	70	22	926931-941674	PHAGE_Entero_N15_NC_001901, ...
3	15.5Kb	incomplete	60	21	1216453-1231993	PHAGE_Stx2_converting_1717_NC_011357, ...
4	30.9Kb	intact	110	43	2013882-2044865	PHAGE_Rhizob_vB_RglS_P106B_NC_023566, ...
5	18.7Kb	intact	110	28	2694534-2713283	PHAGE_Rueger_DSS3_P1_NC_025428, ...
6	15.1Kb	questionable	70	18	2990477-3005586	PHAGE_Tetras_SI1_NC_020861, ...

This is an example of what the output from PHAST looks like. The red rows indicate an intact phage, green rows indicate a questionable phage, and the gray rows indicate an incomplete phage.

Sinorhizobium Strain	Number of Intact Prophages	Size (Kb)
A321	1	48.3
HM007-12	1	33.2
KH36b	1	22.7
KH36d	1	13.7
KH53a	2	30.9, 18.7
M10	1	38.6
M102	1	59.7
M161	3	19.2, 21.7, 43.1
M22	2	44.5, 22.5
M210	1	46.2
M270	2	18, 36.3
N6B7	3	14.9, 19.7, 27.6
WSM419	1	32.1

7

Results: UV Induction

- Phage typing of M102 and KH36b exposed to UV light for 40s lysates are positive for plaques

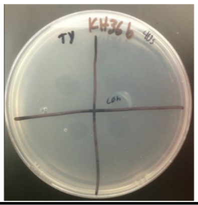

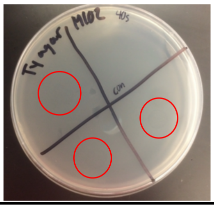

8

Results: Mitomycin Induction

- All plates from mitomycin induction appeared similar to the control plates

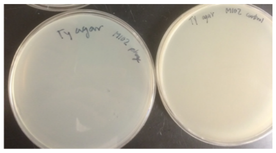

9

Future Directions

- Use restriction enzymes to figure out the sequence of the phage genome
- Investigate phage genome for genes important to bacteria
- Send for TEM images
- Obtain a high titer for KH36b phage

10
