# Supplementary material for: In silico Phage Hunting: Bioinformatics Exercises to Identify and Explore Bacteriophage Genomes
Source: Front Microbiol. 2020 Sep 17;11:577634. doi: 10.3389/fmicb.2020.577634 (PMC7533560; doi:10.3389/fmicb.2020.577634)
Supplement: Supplementary file 3 [file Data_Sheet_3.PDF]

Pre-test/Post test
Name:___________________________________

1) **LO1-**The structure of many bacteriophages contains a tail. The function of the tail is to:

    A. To pass the nucleic acid to the host during infection
    B. To support the head of the bacteriophage
    C. To bind to host's receptors for attachment
    D. To prevent bacteriophage degradation after host infection

2) **LO2** - A/an _______ is specifically able to have a long-term relationship with the host known as lysogeny.

    A. Temperate virus
    B. Adsorbed virus
    C. Virion
    D. RNA phage
    E. DNA phage

3) **LO5-** Which of the following public databases can be used to retrieve bacterial genome sequences
    A. Protein Databank (PDB)
    B. BLAST
    C. PubMed
    D. NCBI Genome
    E. GenBank

4) **LO2-** The process by which phage reproduction is initiated in lysogenized culture is called

    A. Infection
    B. Integration
    C. Repression
    D. Induction
    E. Enhancement

5) **LO1-** In many bacteriophages, the DNA genome is contained in the head, and binding to the host is facilitated by attachment of the:

    A. Capsid
    B. Neck
    C. Tail fibers
    D. Envelope
    E. Tegument proteins

6) **LO2**-Which of the following steps is NOT part of the life cycle of a lytic phage?

A. Phage DNA is injected into the bacterial cell.
B. The phage DNA integrates into the bacterial chromosome.
C. Many copies of phage DNA are made.
D. The phage DNA is transcribed, and the resulting mRNA is translated to make capsid proteins.
E. All of the above steps are part of the life cycle of a lytic phage.

7) **LO2-**The integrated phage genome is called a(n):

A. temperate
B. lytic
C. lysogen
D. prophage
E. oncogene

8) **LO2-**What happens to the virus protein coat when a bacterial cell is infected?

A. It enters the host cell with the viral genome.
B. It remains on the outside of the host cell.
C. It enters the host cell separately from the viral genome.
D. It is released to attach to and inject another host cell.
E. It becomes part of the host cell membrane.

9) **LO1**-Most bacteriophage genomes contain:

A. Double stranded DNA
B. Single stranded RNA
C. Single stranded DNA
D. Double Stranded RNA
E. Both RNA and DNA

10) **LO1-**The basic structure of a virus contains:

A. a nucleic acid
B. a cell wall
C. a protein coat
D. both a and b
E. both a and c

11) **LO2-** In the attachment step of phage replication

A. attachment is nonspecific
B. carbohydrate receptors in the phage tail attach to specific receptors on the bacterial cell wall
C. protein fibers in the phage tail attach to specific receptors on the bacterial cell wall
D. phage DNA exits the protein coat and attaches to receptors on the bacterium

12) **LO2-** In phage replication

A. only the phage nucleic acid enters the bacterial cell
B. the whole phage particle enters the cell and the protein outer coat is removed
C. multiple phage particles enter the same host bacterial cell at the same time
D. only the phage protein enters the bacterial cell

13) **LO3-** Phylogenetic trees can be constructed using data from:

A. Fossils
B. Morphological data
C. DNA sequences
D. Protein sequences
E. All the above

14) **LO2-** The correct order of the stages for phage replication are:

1= assembly
2 = attachment
3 = transcription/replication
4 = release
5 = penetration

A. 5, 2, 3, 1, 4
B. 2, 5, 3, 1, 4
C. 2, 5, 1, 3, 4
D. 5, 2, 1, 3, 4

15) **LO3-** The term "rooted phylogenetic tree" refers to:

A. A phylogenetic tree that dates back to the origin of planet Earth.
B. A phylogenetic tree in which the base or "trunk" represents the common ancestor of all taxa.
C. A phylogenetic tree with no branches and limited number of taxa
D. A phylogenetic tree that indicates uncertainty about the evolutionary relationships of the taxa depicted on the tree.
E. A phylogenetic tree that only illustrates gene duplication events

Use this figure to answer questions 16-18

16) **LO3-** A common ancestor for the species represented by C and E could be at position:

A. 1
B. 2
C. 3
D. 4
E. 5

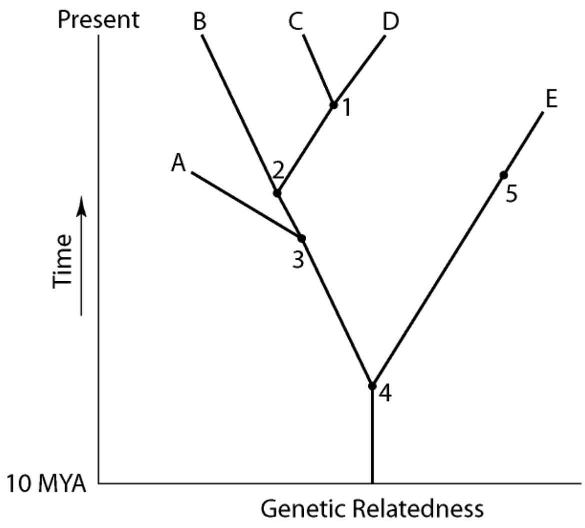

17) **LO3**- The two species that are most closely related to each other are

A) A and B.
B) B and C.
C) C and D.
D) D and E.
E) E and A.

18) **LO3**- If this evolutionary tree is an accurate representation of relatedness, which of the following statement (s) are true?

1. Species A is the direct ancestor of both species B and species C.
2. The species present at position 3 is ancestral to C, D, and E.
3. If all species depicted here make up a taxon, this taxon is monophyletic.
4. The last common ancestor of species B and C occurred more recently than the last common ancestor of species D and E.
5. The last common ancestor of species B, C and D is present at position 2.

A) 2 and 5
B) 1 and 3
C) 3,4, and 5
D) 2, 3, and 4
E) 1, 2, and 3

19) **LO3**- The following data shows four amino acids found across three microbial genera.

Species Amino acid sequence

Bacillus: Arg - Asp - Cys - Arg
Salmonella: Lys - Lys - Cys - Arg
Escherichia: Lys - Lys - Cys - Arg

Which phylogenetic tree best represents the information in the table above?

A. *Salmonella Bacillus Escherichia*

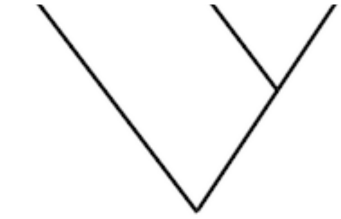

B. *Bacillus Salmonella Escherichia*

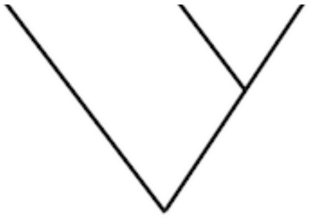

C. *Bacillus Salmonella Escherichia*

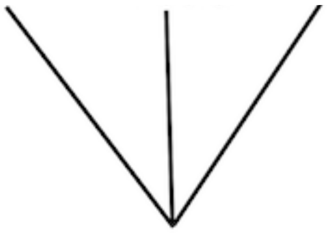

D. *Escherichia Bacillus Salmonella*

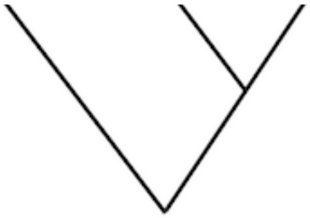

21) **LO4**- The PHASTER tool provides information about predicted bacteriophage proteins in genomes and their sequence similarity to known viral proteins. Which of the following parameter (s) can be used to assess the reliability and quality of the prediction?

A. % GC content
B. Number of amino acids in the protein sequence
C. E- value
D. % GC content and E- value
E. None of the above

22) **LO4-** What type of information or raw data is needed to perform a computational search for phages in microbial genome sequences?

A. The chromosomal and plasmid DNA sequences of the bacterium of interest
B. The protein sequences of the bacterium of interest
C. Only the chromosomal DNA sequence of the bacterium of interest
D. The protein and chromosomal DNA sequences of the bacterium of interest
E. None of the above

23) **LO2-**A lysogenic bacteriophage is induced into a lytic cycle through which of the following events?

A. In response to an increase in extracellular ion concentration
B. In response to an environment factor that damages DNA
C. In response to an increase in virions in the extracellular environment
D. All of the above

24) **LO1**- The genetic material of the phage is protected by a protein coat known as:

A. Capsid
B. Envelope
C. Collar
D. Tail
E. Prion

25**) LO4-** What type of information can usually be retrieved from in-silico phage hunting tool? Use your experience with the PHASTER tutorial to answer this question.

A. Genome sequences of predicted phages
B. Sequences of predicted phage proteins
C. % Sequence homology to known phages
D. Number of predicted phages in a genome
E. All the above

26) **LO2**-Which of the following can cause a phage in the lysogenic stage to revert to the lytic stage?

A. lack of nutrients
B. ultraviolet light
C. a competing phage
D. darkness
E. an electrical charge

27) **LO4-** Which of the following is <u>not</u> a criterium used by Bioinformatics tools to detect phages in bacterial genome sequences?

A. Variation in % G+C content
B. Size of the bacterial genome
C. Sequence comparisons to databases of known phages
D. Presence of phage attachment sites
E. Number of tRNA genes

28) **LO4-** A bioinformatics search using PHASTER predicted three complete prophage regions in the chromosome of *Escherichia coli.* What computational experiment could you do to determine if these phage regions encode bacterial toxins?

A. Compare the size of the prophage regions
B. Compare the %GC of each region
C. Retrieve the phage genome sequences and create a phylogenetic tree
D. Retrieve the prophage protein sequences and do a BLASTp search
E. None of the above

29) **LO1-**The main criteria used to group viruses are structure, chemical composition, and what other characteristic?

A. Type of capsid
B. Overall viral size
C. Area of host cell in which the virus multiplies
D. Similarities in genetic makeup
E. Presence and type of envelope

30) **LO5-** You were assigned to work on a research project that requires retrieval of bacteriophage genome sequences. Which of the databases listed below would be the best resource to get these data?

A. NCBI GenBank
B. NCBI Genome_Viruses
C. NCBI Genome_Microbes
D. PhageWeb
E. The Actinobacteriophage Database

31) **LO1-**At the very minimum, a virus must carry genes for all but which of the following functions?

A. Mature virus packaging
B. Transcription of RNA to DNA
C. Viral capsid synthesis
D. Host regulation
E. Synthesis of viral genetic material

32) **LO5-** Which of the following databases is the best resource for retrieval of viral protein sequences?

A. NCBI_GenBank
B. NCBI_Protein
C. NCBI Genome_Viruses
D. Protein Databank
E. PHASTER
