# Supplementary material for: In silico Phage Hunting: Bioinformatics Exercises to Identify and Explore Bacteriophage Genomes
Source: Front Microbiol. 2020 Sep 17;11:577634. doi: 10.3389/fmicb.2020.577634 (PMC7533560; doi:10.3389/fmicb.2020.577634)
Supplement: Supplementary file 4 [file Data_Sheet_4.PDF]

Bio5550: Microbiology

Laboratory Project #1: *In Silico* Phage Hunting: Bioinformatics Tools to Detect and Explore Bacteriophages Genomes

Introduction

Viruses are the most abundant biological entities on earth, with an estimated 10^{31} existing in the biosphere [4]. Bacteriophages are a type of virus that infect and replicate their genetic material inside bacteria or archaea. The ability to infect bacteria, as well as having an easily manipulated genome, make phages viable for use in biotechnology as well as being a useful tool in uncovering evolutionary patterns of bacteria. Phages have been used as pharmacological vectors to deliver drugs, agricultural control agents, an alternative to antibiotics, and tools for screening libraries of protein and peptide sequences. Phages are also critical agents in gene mobilization and contribute to the coevolution of bacteria, since many bacterial chromosomes contain sequences of prophage genetic material. In some bacterial genomes, as much as 20% can consist of prophage DNA [1].

Despite their important biotechnical applications and evolutionary significance, little progress has been made in terms of comparative genomic analysis studies. To date, there exists around only 500 sequenced genomes of phages that infect the genus *Escherichia* compared to the 66,000 Escherichia bacterial genomes that are publicly available; and this is one of the most commonly studied genera of bacteria [4]. Even more research is needed in exploring phages that infect less commonly studied genera, such as rhizobia bacteria, which form a symbiotic relationship with legumes in order to fix nitrogen [3]. These bacteria play a crucial role in promoting plant growth and have been shown to be a biological alternative to industrial fertilizers [5]. In short, the world of phages needs to be explored on a deeper and broader level in order to fully maximize the biotechnological applications and enhance the evolutionary understanding of these important viruses. Recent advancements in computer technology have allowed this previously inaccessible world to be opened with the use of data mining and bioinformatics.

The use of data mining and bioinformatics is necessary in order determine the abundance and diversity of phage species, determine genetic relationships, and conduct analysis on the function of individual genes. From an evolutionary standpoint, a more precise role of phages that infect certain types of bacteria can be determined since phages have been shown to select for specific genotypes to appear in a population of bacteria [3]. Additionally, this type of research allows for the classification of existing phages. Data mining on sequenced genomes is an important component to further understanding about phage host range, life cycle, biochemical mechanisms, and determine genetic relationships to other phages.

Bacteriophages come in different sizes and shapes but most of them have the same basic features: a head or capsid and a tail. The head contains the viral genetic material and the tail is a hollow tube through which the nucleic acid passes through when the bacteriophage infects a host cell. In some phages, the tail is surrounded by a contractile protein sheath, which contracts during infection of bacterial host. The baseplate is a structure at the end of the tail that holds one or more tail fibers attached to it. The long tail fibers are used by the bacteriophage to attach itself to the bacterium and the virus then inserts (Figure 2).

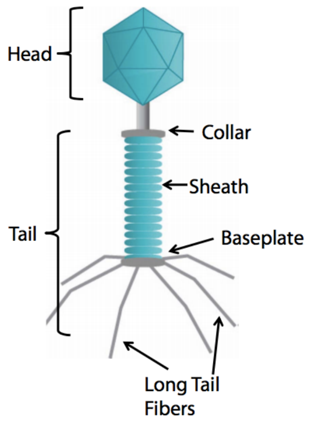

Figure 2. Structure of a bacteriophage

Soon after the discovery of bacteriophages, scientists observed that upon infection, certain phages could halt bacterial metabolism, replicate and kill the host by cell lysis. In contrast, other bacteriophages were partially- lytic and integrated into the host chromosome after infection. These types of phages are known as “temperate”, since they tempered (moderated) their destruction of the host population. Bacterial viruses inserted in their host chromosome are known as “prophages”, while the bacteria containing them are referred to as lysogens. These bacteria can induce their lysis upon exposure to stress or harmful conditions. The process by which prophage production and host lysis are activated in a lysogen is known as induction.

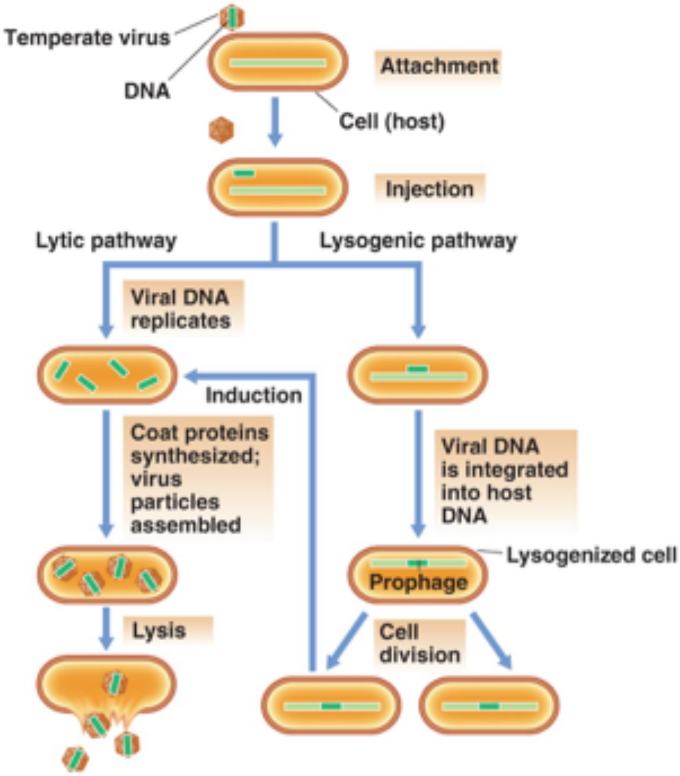

Figure 3. Lytic and Lysogenic Cycle of Phage Lambda (**λ**)

Bacteriophage lambda (**λ**) is a temperate bacteriophage and can undergo cycles of lysis and lysogeny after infecting its host (Figure 3). When lambda is lysogenic, it integrates into to the host genome at specific sites known as lambda attachment sites (*att* **λ**). Integration requires a special enzyme known as an “integrase”, which recognizes the *att* sites in both the phage and the host DNA. Once integrated in the host DNA, the phage genome is replicated along with the bacterial chromosome.

During the lytic cycle, bacteriophages recognize the host and attach to specific cell surface receptors using the tail fibers. For example, bacteriophage T4 infects Gram-negative bacteria by recognizing the lipopolysaccharide in the outer membrane and using it for fiber attachment. The virus then injects its DNA into the host cell through a process that involves contraction of the tail's sheath and penetration of the inner tail tube into the outer membrane and cell wall of the host. The inner tail tube extends into the cytoplasmic membrane of the host and the phage head releases the DNA under packing pressure delivering it into the cytoplasm. Following genome injection, the phage highjacks the cellular machinery and starts making its own proteins. The early and middle genes are transcribed first, allowing the synthesis of nucleases that cleave the host's genome, DNA polymerase and other proteins needed for further phage replication. Late mRNAs encode for the structural proteins necessary to make the head and tail components of the virus and for any other enzymes needed for the release of mature virions from the host. The viral DNA is packed into the heads and the head are assembled onto tails. The mature viral particles lyse the bacterial cells and are released into the environment where they remain until finding a new host (Figure 4).

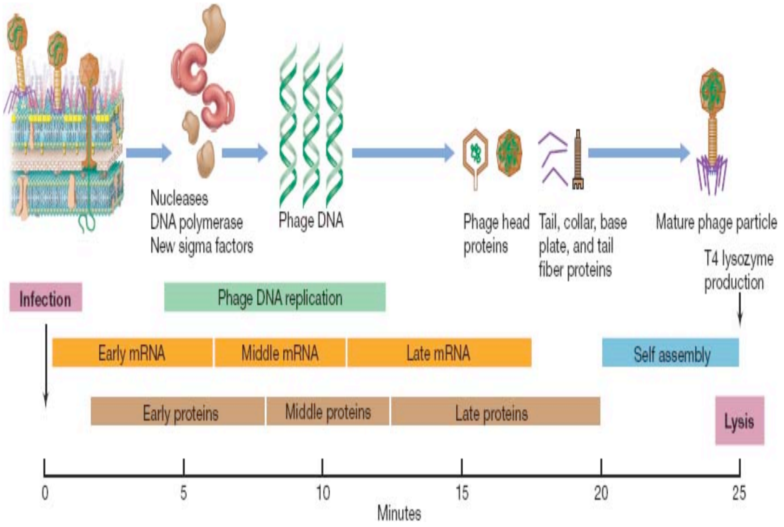

Figure 4. Events during bacteriophage T4 infection

Objectives

As part of Bio5550 (Microbiology), we will work on a multi-week laboratory project to investigate and learn about the biology of bacteriophages present in diverse bacteria, including species like *Sinorhizobium* which fix nitrogen when forming a symbiotic relationship with the model legume, *Medicago truncatula*. We will explore bacteriophages in the genomes of various strains of *Escherichia coli*, including pathogenic and commensal isolates.

This project aims to elucidate the abundance and diversity of phages that infect common bacteria by performing data mining of bacterial chromosome sequences available from GenBank. PHASTER, a powerful program which can determine the exact location of a phage on a bacterial chromosome, will be used in order to compare intact protein sequences between phage species, allowing for determinations to be made about the abundance of phages in bacteria. Clustal Omega and MAFFT will also be used to align DNA and protein sequences in order to generate phylogenetic trees. The bioinformatics analyses will also aim at comparing the protein sequences of "phage integrases". These proteins are involved in viral DNA insertion into the host chromosome. Bioinformatics comparisons of their sequences are one of the criteria used for bacteriophage classification.

During the final weeks of the project, laboratory teams will perform a comprehensive review of literature to come up with an original hypothesis about bacteriophages. Next, you will design an experimental plan to test the hypothesis posed using computational tools, wet bench experiments or a combination of both. All teams will report their finding by doing a final presentation during the last week of the project.

The projected aims of this exercise are as follows:

- Utilize data mining and bioinformatic analysis to determine the abundance of intact bacteriophages in the genomes of the diverse bacteria
- To compare the genome sequences of bacteriophages in order to identify conserved genetic elements and describe overall structural organization
- To identify and annotate the protein sequences encoded in bacteriophage genomes and use these data to generate phylogenetic trees and investigate evolutionary relationships amongst phages.

References

1. Arndt, D., Marcu, A., Liang, Y., & Wishart, D. S. (September 2017). PHAST, PHASTER, and PHASTEST: Tools for finding prophage in bacterial genomes. Briefings in Bioinformatics, 1-8.

2. Hatfull, F. G. (October 2008). Bacteriophage Genomics. Curr Opin Microbiol, 11(5), 1-10.

3. Santamaria, I. R., Bustos, P., Sepulveda-Robles, O., Lozano, L., Rodriguez, C., Fernandez, L. J., . . . & Gonzales, V. (January 2014). Narrow-Host-Range Bacteriophages That Infect Rhizobium etli Associate with Distinct Genomic Types. Applied and Environmental Microbiology, 80(2), 446-454.

4. Sazinas, P., Redgwell, T., Rihtman, B., Grigonyte, A., Michniewski, S., Scanlon, J. D., Hobman, J., & Millard, A. (December 2017). Comparative Genomics of Bacteriophage of the Genus Seuratvirus. Genome Biol. Evol., 10(1), 72-26.

5. Vessey, J. K. (August 2003). Plant growth promoting rhizobacteria as biofertilizers. Plant and Soil, 225(2), 571-586.
