# Supplementary material for: In silico Phage Hunting: Bioinformatics Exercises to Identify and Explore Bacteriophage Genomes
Source: Front Microbiol. 2020 Sep 17;11:577634. doi: 10.3389/fmicb.2020.577634 (PMC7533560; doi:10.3389/fmicb.2020.577634)
Supplement: Supplementary file 5 [file Data_Sheet_5.PDF]

Supporting File S5. Rubric used to Evaluate Student's Final Presentations

| **Skills Related to the Design and Oral Presentation of Research Projects** | **Does Not Meet Expectations**<br><br>**1** | **Approaches Expectations**<br><br>**2** | **Meets Expectation**<br><br>**3** | **Exceeds Expectation**<br><br>**4** |
|---|---|---|---|---|
| **Introduction** | -The introduction does not talk about the bacteriophages and their relevance to biology<br>-The key events in the life cycle of viruses are not mentioned<br>-The rationale for the proposed research project is missing<br>-The hypothesis for the proposed research is missing or only implied<br>-The introduction does not include any citations or scholarly references | -The introduction includes only a few aspects of bacteriophages and their relevance to biology<br>-The description of the events in the life cycle of phages are vague or incomplete<br>-The rationale for the proposed research project is not clear or illogical according to previously published research<br>- The introduction does not include any citations or scholarly references<br>-The hypothesis is too broad, vague, does not follow from background, OR only verbal | -The introduction is generally ok, but some aspects of the bacteriophage structure and function are unclear, misleading, OR too general<br>- Explanation of key events leading to the establishment of symbiosis is good but some aspects are incorrect, rushed or irrelevant<br>-The research leading to the proposed project is explained briefly or is vague and supported by few (less than three) scholarly references<br>-The hypothesis is somewhat unclearly stated or the connection to background material unclear | -The introduction states the fundamental structure and relevance bacteriophages to biology and medicine<br>-Explains the key events of the lytic and lysogenic cycle of phages<br>-The research leading to the proposed project is explained in detail and supported by three or more scholarly references<br>-The hypothesis for the proposed research is clearly stated and logical based on previous research |
| **Methods** | -Overall experimental approach totally unclear<br>-Level of detail is insufficient<br>- The theory and biological basis of the techniques and tests used in the experimental assays are missing<br>-The experimental design lacks controls and experimental replicas for all assays<br>- The relevance of computational tools to meet the goals of the project is missing | -Overall approach very disorganized OR too long/short<br>-Level of detail often insufficient or excessive (e.g. step-by-step protocol)<br>- The theory and biological basis of the techniques and tests used in the experimental assays is too general or vague<br>-The experimental design has incorrect controls OR improper number of replicas for all assays | -Overall approach somewhat disorganized OR too long/short<br>-Level of detail occasionally excessive OR insufficient<br>- The theory and biological basis of the techniques and tests used in the experimental assays is complete but some aspects are incorrect or unclear<br>-The experimental design includes appropriate controls and number of experimental replicas for some but not all assays. | -Overall approach clear, organized, & concise<br>-Level of detail is appropriate (e.g. describes sample sizes, variables, replicates, controls, specialized procedures)<br>-The theory and biological basis of the techniques and tests used in the experimental assays are accurately explained<br>-The experimental design included proper controls and experimental replicas for all assays. |

This rubric is an adaptation of the oral presentation rubric created by the Department of Biology at Hamline University. All the standards and descriptions of assessments were created by the biology faculty. They have kindly agreed to let other faculty members modify and use this rubric for the assessment of oral research presentations in different courses.

Supporting File S5. Rubric used to Evaluate Student's Final Presentations

| | | | | |
|---|---|---|---|---|
| | | The relevance of computational tools to meet the goals of the project is briefly mentioned but not explained in detail | The relevance of computational tools to meet the goals of the project is explained briefly and occasionally lack accuracy | The relevance of computational tools to meet the goals of the project is explained accurately and in great detail |
| **Skills Related to the Design and Oral Presentation of Research Projects** | **Does Not Meet Expectations**<br><br>**1** | **Approaches Expectations**<br><br>**2** | **Meets Expectations**<br><br>**3** | **Exceeds Expectations**<br><br>**4** |
| **Results** | -Data illustrated improperly; most labels missing<br>-Labels not described<br>-Trends always rushed, unclear or missing<br>-Data analysis was not appropriate<br>-Retrieval procedures and interpretation of bioinformatics results are unclear or missing | -Data illustrated with too few OR too many figs/tables; labels or figs inappropriate<br>-Labels usually described incompletely or excessively<br>-Trends often rushed, unclear, or missing<br>-Data analysis was often unclear<br>-Retrieval procedures and interpretation of bioinformatics results are limited and vaguely explained | -Data illustrated appropriately but some labels unclear<br>-Labels sometimes described incompletely or excessively<br>-Trends occasionally unclear or missing<br>-Data analysis occasionally unclear or missing some information<br>-Retrieval procedures and interpretation of bioinformatics results are mostly accurate and clearly explained | -Data illustrated in appropriate tables &/or figs (e.g. graphs, photos, diagrams) with meaningful labels (not acronyms)<br>-Labels of tables/figs are concisely and clearly explained & pointed out<br>-Trends clearly & concisely expressed in take-home message for each table or fig<br>-Data analysis appropriate & clear; if statistical analysis present, includes name of test & P-value<br>Retrieval procedures and interpretation of bioinformatics data are explained accurately and in detailed |
| **Discussion** | -Results interpretation not related to hypothesis<br><br>-Explanation of results missing<br><br>-Conclusion missing | -Results interpretation unclear about whether or not hypothesis was supported<br><br>-Explanation of results was unclear, incorrect, OR not adequately supported by references | -Results interpretation somewhat unclear about whether or not hypothesis was supported<br><br>-Explains results, but with some logic flaws or misinformation, references poorly integrated into argument OR does not place study into context of field | -Results interpretation clearly indicates whether or not hypothesis was supported<br><br>-Explains results in the context of the field, supporting points with references, noting areas of agreement & disagreement |

This rubric is an adaptation of the oral presentation rubric created by the Department of Biology at Hamline University. All the standards and descriptions of assessments were created by the biology faculty. They have kindly agreed to let other faculty members modify and use this rubric for the assessment of oral research presentations in different courses.

Supporting File S5. Rubric used to Evaluate Student's Final Presentations

| | | | | |
|---|---|---|---|---|
| | | -Conclusion attempted but unclear or incorrect | -The conclusion is somewhat vague or wordy, or at inappropriate location | -Conclusion clearly stated as take-home message at end of discussion OR on final conclusion slide |
| **Skills Related to the Design and Oral Presentation of Research Projects** | **Does Not Meet Expectations**<br><br>**1** | **Approaches Expectations**<br><br>**2** | **Meets Expectations**<br><br>**3** | **Exceeds Expectations**<br><br>**4** |
| **Group participation** | -Members of the group were missing or refused to participate in the presentation<br><br>-One or more members of the group refused to answer questions from the audience or the instructor | - All members of the group participated in the presentation<br><br>- One person did most of the talking and answered all the questions preventing other students from contributing to the presentation | -All members of the group actively participated in the presentation<br><br>- Only a few group members were able to answer questions from the instructor and the audience | -All members of the group actively and equally participated in the presentation<br><br>-All group members were able to answer questions from the instructor and the audience |
| **Style** | -Slides have much extraneous, missing or unreadable, information | -Slides usually unclear (e.g. too much text) or not well-explained | -Slides usually clear and well-explained, may forget to point to important content | -Slides clear, easy to read and understand (bulleted, minimal text, simple diagrams). Explains slides by pointing to important content |

This rubric is an adaptation of the oral presentation rubric created by the Department of Biology at Hamline University. All the standards and descriptions of assessments were created by the biology faculty. They have kindly agreed to let other faculty members modify and use this rubric for the assessment of oral research presentations in different courses.
