# Supplementary material for: In silico Phage Hunting: Bioinformatics Exercises to Identify and Explore Bacteriophage Genomes
Source: Front Microbiol. 2020 Sep 17;11:577634. doi: 10.3389/fmicb.2020.577634 (PMC7533560; doi:10.3389/fmicb.2020.577634)
Supplement: Supplementary file 6 [file Data_Sheet_6.PDF]

| BIOINFORMATICS COMPETENCIES | EXERCISES TO INCORPORATE BIOINFORMATICS COMPETENCIES |
|---|---|
| C1. **EXPLAIN THE ROLE OF COMPUTATION AND DATA MINING IN ADDRESSING HYPOTHESIS-DRIVEN AND HYPOTHESIS- GENERATING QUESTIONS WITHIN THE LIFE SCIENCES.** | • Formulate hypotheses regarding the abundance of phages in different bacterial species.<br>• Examine genome sequences to investigate genes and proteins essential for phage structure and function.<br>• Explain traditional wet bench experiments to isolate phages to computational phage-finding tools. |
| C2. **SUMMARIZE KEY COMPUTATIONAL CONCEPTS, SUCH AS ALGORITHMS AND RELATIONAL DATABASES, AND THEIR APPLICATIONS IN THE LIFE SCIENCES.** | • Describe the criteria used by PHASTER to identify bacteriophages in bacterial genomes.<br>• Explain how using phage finding programs and databases can help answer biologically relevant questions. Use NCBI genome to retrieve accession numbers and run the PHASTER, the phage identification tool. |
| C3. **APPLY STATISTICAL CONCEPTS USED IN BIOINFORMATICS.** | • Use E-values, percent identity and gene coverage to evaluate the results of BLAST searches.<br>• Use E-values to identify the closest homologs to phage proteins of known function. |
| C4. **USE BIOINFORMATICS TOOLS TO EXAMINE COMPLEX BIOLOGICAL PROBLEMS IN EVOLUTION, INFORMATION FLOW, AND OTHER IMPORTANT AREAS OF BIOLOGY.** | • Create DNA and protein sequence alignments to compare bacteriophages<br>• Use information generated by bioinformatic searches to create graphs illustrating the abundance of phages in different bacterial species.<br>• Use bioinformatics tools such as CLUSTA omega, MAFFT and iTOL to investigate phylogeny and evolutionary relationship amongst bacteriophages. |
| C5. **FIND, RETRIEVE, AND ORGANIZE VARIOUS TYPES OF BIOLOGICAL DATA.** | • Use the appropriate NCBI databases to find and retrieve genomes, DNA and protein sequences.<br>• Retrieve sequences from various databases. Organize genome, gene and protein sequences. Convert sequences to the appropriate format for BLAST searches and alignments. |
