# Supplementary material for: In silico Phage Hunting: Bioinformatics Exercises to Identify and Explore Bacteriophage Genomes
Source: Front Microbiol. 2020 Sep 17;11:577634. doi: 10.3389/fmicb.2020.577634 (PMC7533560; doi:10.3389/fmicb.2020.577634)
Supplement: Supplementary file 7 [file Data_Sheet_7.PDF]

Resources for Instructors

1. Bioinformatics Tools to Study Bacteriophages

Resource Name	URL	Description
PHASTER	https://phaster.ca/	Tool to search for bacteriophages in bacterial genomes
VIRFAM	http://biodev.cea.fr/virfam/	Detection and classification of bacteriophages using head and tail proteins
MVP Database	https://mvp.medgenius.info/home	A microbe-phage interactions database. Allows identification of phages sequences and potential bacterial hosts
PhiSiGns	http://phisigns.sourceforge.net/	A tool to identify signature genes in phages and design primers
SEA PAHGES	https://seaphagesbioinformatics.helpdocsonline.com/home	Bioinformatics guide from the SEA PHAGES Programs
CRISPRFINDER	https://crispr.i2bc.paris-saclay.fr/Server/	A program to find CRISPRs in DNA sequences
iTOL	https://itol.embl.de/	Tool to visualize, store and annotate phylogenetic trees
MAFFT	https://mafft.cbrc.jp/alignment/server/	Multiple sequence alignment program for large data sets. Creates alignments and phylogenetic trees
CLUSTAL OMEGA	https://www.ebi.ac.uk/Tools/msa/clustalo/	Multiple sequence alignment program. Creates alignments and phylogenetic trees

2. **Accession numbers for PHASTER Exercises**. The accession numbers in the table below can be used to run PHASTER and compare the number of phages in diverse bacterial species.

Strain	Accession Number
Sinorhizobium meliloti AK83	NC_015590.1
Sinorhizobium medicae WSM419	NC_009636.1
Rhizobium leguminosarum bv. trifolii WSM1689	NZ_CP007045.1
Mesorhizobium sp. B7 complete genome	NZ_CP018171.1
Bradyrhizobium oligotrophicum S58	NC_020453.1
Escherichia coli str. K-12 substr. MG1655	NC_000913.3
Escherichia coli O83:H1 str. NRG 857C	NC_017634.1
Escherichia coli UMN026 chromosome	NC_011751.1
Escherichia coli IAI39 chromosome	NC_011750.1
Salmonella enterica subsp. enterica serovar Chester str. ATCC	NZ_CP019178.1
Salmonella enterica subsp. enterica serovar Minnesota str. ATCC .4592393	NZ_CP019184.1
Shigella flexneri 2a str. 301	NC_004337.1|
Bacillus cereus ATCC 14579	NC_004722.1
Bacillus subtilis BSn5	NC_004722.1
Bacillus anthracis str. Ames	NC_003997.3
Bacillus anthracis str. Turkey32	NC_003997.3
Bacillus thuringiensis serovar konkukian str. 97-27	NC_003997.3
Bacillus subtilis PY79	NC_003997.3
Bacillus horikoshii strain 20a	NC_003997.3

3. Integrase Protein Sequences for Alignments (PhageIntegrases_Set1)

```
>1_EDL933
MLNGYIDEGKAASAKLIRSTLSDAFREAIAEGHITTNPVAATRAAKSEVRRSRLTADEYL
KIYQAAESSPCWLRLAMELAVVTGQRVGDLCEMKWSDIVDGYLYVEQSKTGVKIAIPTTL
HVDALGISMKETLDKCKEILGGETIIASTRREPLSSGTVSRYFMRARKASGLSFEGDPPT
FHELRSLSARLYEKQISDKFAQHLLGHKSDTMASQYRDDRGREWDKIEIK
>2_EDL933
MRKTSVAKVWQNYELEKAKLHNIMTVAKLWHMFMDSPAFTELAPRTQKDYRQHQKALLMV
FGKVLADNVKTEQVRIFMDKRGLESKTQANHELASLSRVYGWGYERGYVKNNPCKGVRKF
SLKARTVYITDEQYAAIYAEAIPQLRIAMEISYLCAARLGDVLELKWQDIMDKGIYIEQN
KTGTKQIKEWSPRLRTAIQLARNVSSCTCEYVINTTKGGKVIAKTLNNWWNQAKRAAEQK
VGVPFGCNFHDIKAKGISDYEGSSRDKQIFSGHKTENQVLIYDRKTKITPTLDLPLVVSK
>3_EDL933
MANSAYPAGVENHGGKLRITFKYRGKRVRENLRVPDTPKNRKIAGELRASVCFAIRTGTF
DYADRFPDSPNLKLFGLVKKDITVGELAQKWLTLKAMEIGSNALNRYQSVMKNMLPRLGP
GRLASSITKEDLLFIRKDLLTGEKGSRKTSTSRKGRTVPTVNYYMTTTAGMFSFAAENGY
LEKNPFNSITPLRKSKPVPDPLTRDEFSRLIDACHHQQTKNLWTVAVFTGMRHGEIAALA
WEDIDLKAGTITVRRNFTKIGDFTLPKTDAGTNRVIHLLAPAIEALKNQAMLTRLSRQHQ
ITVQLREYGRTILHECTFVFCPQIVRKNHKAGINYAVSSIGATWDSAIKRAGIRSRKAYQ
SRHTYACWALSSGANPTFIASQMGHSSASMVYNVYGAWMPECSVTQVAMLNNVLNARAPD
VPQSDQEDEIKLYFSK
>4_EDL933
MSPRPRKNSTDVAGLYEKFDRRTGRVYYQYKNPVTGKFHGLGTDKGKAEKIASTANQRIA
AAEAEYFMRKIDESPSATKRRGIRLKAWVDRYLKIQDTRLKNGDIAATTHKEKTRMAAYL
VSRLGNHPLKELEVRDFALILDEWLDKDMVSTARVNRGLWVDIYKEAQHAGEVPPGWNPP
EATRKPIPKVTRARLTMEDWQKIYNATPEKHFIRNAMLLAIVTGQRRDDICHMRFSDVWN
EHLHITQGKTGMRLALPLTLRCDAIGITLKEVIDGCRDRILSPYLIHSRHQKQPKPMSKD
NLSDYFAKARDLAGVIPPAGKTPPTFHEQRSLSERLYRAQGIDTKTLLGHKVQATTDRYN
DTRGQEWVKLVI
>5_EDL933
MAASPRSHKISIPNLYCKLDKRTGKVYWQYKHPLSGRFHSLGTDENEAKQVATEANTIIA
EQRTRQILSVNERLERMKGRRSDITVTEWLDKYNSIQEDRLQHNELRPNSYRQKGKPIRL
FREHCGMQHLKDITALDIAEIIDAVKAEGHNRMAQVVRMVLIDVFKEAQHAGHVPPGFNP
AQATKQPRNRVNRQRLSLPEWQAIFDSVSRRQPYLKCGMLLALVTGQRLSDICNLKFSDI
WDDMLHITQEKTGSKLAIPLNLKCDALNITLREVISQCRDAVVSKYLVHYRHTTSQANRG
DQVSANTLTTAFKKAREKCGIKWEQGTAPTFHEQRSLSERLYREQGLDTQKLLGHKSRKM
TDRYNDDRGKDWIIVDIKTA
>6_EDL933
MARPRKYKTDVPGLSPYFDKRNNKVYWRYRHPITGKNHGLGSIDQKLAETIAAEANSRLA
RQQMEQMLSLQEKIISDTGGSSTVTIFLNNYRKIQQERYENGEIKLNTLKQKAAPLRVFD
ERFGTRPLDAITVKDVVSVLEEYKARGHNRMGQIFRKVLIDVFREAQQTGDVPPGFNPAE
SAKKPQVRISRQRLTFDEWMMIYNAAEKDGYFLQRGMLLALMTGQRLSDICKMQFSDIRD
GYLHVEQQKTGTRIAIPLALRCDKLNLTLDDVVSSCRDCVLSPWLLHHHHAKGTAKRGGM
VKPATLTVAFKKARDSVDYNWRANGTPPSFHEQRSLSERLFREQGVDTKILLGHSNQKMT
DIYNDARGKEWKKLVI
>7_EDL933
MSVRKIPSGKWLCECYPYGASGKRIRKQFATKSEALSYERRLMNSRVGDEFQDGSGPRLS
ELIARWFEMYGKTLSSGAERKVKLEAICSRLGDPFASQFDKNMFATYRERRLSGEWNPKG
KKKLSEATVNREQSYLHAVFAELKRLGEWSGENPLTGIRKFREEEKELAFLYVDEIERLL
IACDESRNKDLGVVVRIGLATGARWSEAEGLKQSQVLPGRITFVKTKGKKNRTVPISPQL
QAMLPKKRGALFSPCYEAFDAAIKRAKIELPDGQLTHVLRHTFASHFMMRGGNILVLQKI
LGHSDIKMTMRYAHFAPGHLEAAVELNPFDNRG
>8_EDL933
MGRRRKNPEHEKLPPKVYPNKYSVWKPTSRESVTLTAIEDGLAALWKKYEETVNHRDRAM
TFGRLWEKFLASAYYSELSPRTQKDYLQHQKKLLAVFGKVLADSVKPEHIRRYMDKRGEQ
SKTQANHEKSSMSRVYSWGYERGYVKANPCAGVSKFKAKNRERYVTDKEYQAVLSVAPLP
VFIAMEIAYLCAARVSDVLSLKWEQIGNDGIFIQQGKTGKKQIKAWSPRLQAAIEKAKQL
```

PTSAYVISNQYGNRYMYKGFNEMWVEARNHAGKISGILTDFTFHDLKAKGISDYEGSSRD
KQLFSGHKTEGQVLIYDRKVKVSPTLDVPLPENIPRKYSK

>9_Sakai8
MLNTYVAEGKAVSARVIRSTLVDVFRGAIAEGHVATNPVTTTRAAKSEVRRSRLTANEYV
AIYHAAEHLPIWLRLSMDLAVVTGQRVGDLCRMKWSDINDGHLHIGQSKTGAKIAIPLAL
TIDALDISLVDTLQKCREASSSETIIASTYHEPLSPATVSRYLTKARNASGISFDGDPPT
FHELRSLSARLYRNQIGYKFAQRLLGHKSDSMAAHYRDSRGREWDKIEIG
>10_Unknown
MSNASYPTGVENHGGSLRIWFHYNGKRVRENLGVPDTAKNRKIAGELRTSVCFAIRMGSF
DYAAQFPNSPNLKHFGLGKREITVKALSEKWLDLKKIEICANALNRYQSVIKNMLPMLGE
KKLVSSITKEDLLFVRRDLLTGYQKLSNGKTSSIKGRSVVTVNYYMTTIAGMFQFATDNG
YTSGNPFNGLAPLKKSKVKPDPLTRDEFIRFIEACRHQQTKNLWILAVYTGIRHGELVSL
AWEDIDLKARTITIRRNYTKLGEFTPPKTDAGTGRTIHLVQPAIDALKSQAEMTMLGKQH
SVEVKQREYGRTAVHKCTFVFSPQVTKQQQLSGPHYKVDSIRESWTSILKRAGLRHRKSY
QSRHTYACWSLAAGANPSFIASQMGHTNAQMVFNVYGAWMKDNNHEQIELLNKRLSESVP
CMPHKKAG
>11_O104H4
MDKVKYPTGVENHGGTLRIWFNFKGKRVRENLGVPDTAKNRKIAGELRTSVCFAIRTGSF
DYAAQFPDSPNLQAFGVSKKEITVKELEEKWLDLKRMEISANAFNRYESVARTMVPKIGG
SRLVSSVTKEELLYIRKDLLTGYQNSMKNKAAKGRSVVTVNYYMTTIAGMFQFAADHGYL
EANPFQGIKPLKRARAEPDPLTRDEFIRLIDACRHQQTKNLWSLAVYTGMRHGELVSLAW
EDIDLKAGTITIRRNYTKLGEFTLPKTEASTNRVVHLIQPAISVLKNQAEMTRLGKQYNI
KVQLREYGRSVNHECTFVFNPQVVRKSEQVGFVYKVDSVGDSWETAIKRAGIRHRKAYQS
RHTYACWSLSAGANPSFIASQMGHASAQMVFNVYGAWMTDSNAEQIAMLNQKLTDYVPMM
PHSHQSDTRGLLKSVS
>12_O104H4
MRPEGRKGKRIRKKFKTKSDAVLYERWVLAQQHNNEWKGNSIDRRPLSVLIDLWWKYHGQ
LMKSGHNTRLKLLRLSEAMDDPCVHKLNTTMLTELRVSRIEQGIQPSTINREIGALSAMF
TALISSGHFLNDNPVQGLKGMKVNEREMGYLSKSECVQLLDALAENPDERLAVEILLSTG
ARWGEVAVLEQRRVLHCRITFSKTKNSKNRTVPISESLFEKIKKRGGKLVFPTLDYPLVR
DVIKTVASDVPDGQAVHALRHTFASHFMMNGGNILTLQKILGHAKIQTTMIYAHLAPDYL
QDAVRFNPIAG
>13_O104H4
MSLFRRGEIWYASYSLPGGKRIKESLGTKDKRQAQELHDKRKAELWRVDRLGDMPDVTFE
EACLRWLEEKADKKSIDSDKSRIAFWIEHFEGIRIKDISEAMIYSVISKAYNRKTKERWK
LQVEAALRKGKEPPAYIPKSVSTQTKATHLAMIKAILRAAERDWKWLEKAPVIKIPAVKN
KRVRWLEKEEARRLIDACSDPLKSVVKFALATGLRRSNIINLEWQQIDMQRRVAWVNPED
SKSNRAIGVALNDTACKVLRDQIGKHHRWVFVYTTAARRPDGTMTPSIRKMRLDYNTSWL
TACRRAGIENFRFHDLRHTWASWLIQSGVPLSVLQEMGGWESIEMVRRYAHLAPNHLTEH
ARKIDDILGDDVPNLSHPEVFEDAKKA
>14_O104H4
MARPRKYKTDVPGLSPYFDKRNNKVYWRYRHPITGKNHGLGSIDQKLAETIAAEANSRLA
RQQMEQMLSLQEKIISDTGGSSTVTIFLNNYRKIQQERYENGEIKLNTLKQKAAPLRVFD
ERFGTRRLDAITVKDVVSVLEEYKARGHNRMGQIFRKVLIDVFREAQQTGDVPPGFNPAE
SAKKPQVRISRQRLTFDEWMMIYNAAEKDGYFLQRGMLLALTTGQRLSDICKMQFSDIRD
GYLHVEQQKTGTRIAIPLALRCDKLNLTLDDVVSSCRDCVLSPWLLHHHHAKGTAKRGGM
VKPATLTVAFKKARDSVDYNWRANGTPPSFHEQRSLSERLFREQGVDTNILLGHSNQKMT
DIYNDARGKEWKKLVI
>15_O104H4
MANSAYPAGVENHGGKLRITFKYRGKRVRENLRVPDTPKNRKIAGELRASVCFAIRTGTF
DYAERFPDSPNLKLFGLVKKDITVGELAQKWLTLKAMEISSNALNRYQSVMKNMLPRLGP
GRLASSITKEDLLFIRKDLLTGEKGSRKTSTSRKGRTVPTVNYYMTTTAGMFSFAAENGY
LEKNPFNSITPLRKSKPVPDPLTRDEFSRLIDACHHQQTKNLWTVAVFTGMRHGEIAALA
WEDIDLKAGTITVRRNFTKIGDFTLPKTDAGTNRVIHLLAPAIEALKNQAMLTRLSRQHQ
ITVQLREYGRTILHECTFVFCPQIVRKNHKAGINYAVSSIGATWDSAIKRAGIRSRKAYQ
SRHTYACWALSAGANPTFIASQMGHSSASMVYNVYGAWMPECSVTQVAMLNNVLNARAPD
VPQSDQEDEIKLYFSK

>16_O104H4
MGRRRSHERRDLPPNLYIRNNGYYCYRDPRTGKEFGLGRDRRIAITEAIQANIELFSGHK
HKPLTARINSDNSVTLHSWLDRYEKILASRGIKQKTLINYMSKIKAIRRGLPDAPLEDIT
TKEIAAMLNGYIDEGKAASAKLIRSTLSDAFREAIAEGHITTNPVAATRAAKSEVRRSRL
TADEYLKIYQAAESSPCWLRLAMELAVVTGQRVGDLCEMKWSDIVDGYLYVEQSKTGVKI
AIPTALHVDALGISMKETLDKCKEILGGETIIASTRREPLSSGTVSRYFMRARKASGLSF
EGDPPTFHELRSLSARLYEKQISDKFAQHLLGHKSDTMASQYRDDRGREWDKIEIK
