# Supplementary material for: In silico Phage Hunting: Bioinformatics Exercises to Identify and Explore Bacteriophage Genomes
Source: Front Microbiol. 2020 Sep 17;11:577634. doi: 10.3389/fmicb.2020.577634 (PMC7533560; doi:10.3389/fmicb.2020.577634)
Supplement: Supplementary file 8 [file Data_Sheet_8.PDF]

In Silico Phage Hunting
WS I: Discussion questions to learn about bacteriophages
Name: ________________________

1. Label the following bacteriophage structures and briefly explain their function.

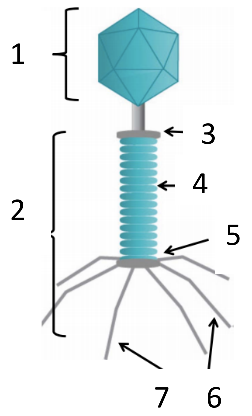

2. Compare and contrast the lytic and lysogenic cycles of temperate phages.

3. How do bacteriophages recognize their bacterial host? Provide two examples?

4. What types of conditions can activate the lytic cycle of a prophage? Explain.

5. Explain the role of bacteriophages in horizontal gene transfer.

6. What would you need to isolate a phage from bacteria or from any natural environment? Explain.

7. Many bacteriophages encode toxins and virulence factors (table below). Discuss how this aspect of phage biology impacts the evolution of new bacterial species. Support your answer with a least one example.

**Bacterium**	**Phage**	**Gene Product**	**Phenotype**
Vibrio cholerae	CTX phage	cholerae toxin	cholera
Escherichia coli	lambda phage	shigalike toxin	hemorrhagic diarrhea
Clostridium botulinum	clostridial phages	botulinum toxin	botulism (food poisoning)
Corynebacterium diphtheriae	corynephage beta	diphtheria toxin	diphtheria
Streptococcus pyogenes	T12	erythrogenic toxins	scarlet fever

In Silico-Phage Hunting
Worksheet II: Database Exploration, Data Retrieval and PHASTER Tool
Name: ___________________________________

Objectives

A. Explore the information available in the NCBI Genomes Database
B. Retrieve microbial genome files to detect the presence of bacteriophages
C. Utilize the PHASTER bioinformatics tool to identify bacteriophages in genome sequences
D. Examine and evaluate the information provided by the PHASTER analysis tool.

Part I. Exploring the NCBI Genome Database

Procedure

The first part of this activity consists of exploring the NCBI Genome database. We will work on locating genome sequences, recording accession numbers, and downloading data files.

1. Use the following URL to access the NCBI genome database: https://www.ncbi.nlm.nih.gov/genome/

2. Go to custom resources and select microbes. This action should take you to this webpage: https://www.ncbi.nlm.nih.gov/genome/microbes/

3. Under the Using Microbial Genomes tab, select Browse microbial genomes. This action should take you to this webpage: https://www.ncbi.nlm.nih.gov/genome/browse#!/prokaryotes/

4. Take a moment examine the information provided in browse microbial genome search page. https://www.ncbi.nlm.nih.gov/genome/browse#!/prokaryotes/

5. Go to the search tab and type *Escherichia coli* MG1655.

6. Examine the column labeled as "replicons". This term refers to DNA molecules that can replicate as a unit; chromosomes and plasmids are examples of replicons. The numbers shown in the replicon column are accession numbers. These are unique identifiers for genomes which can be used to retrieve their DNA sequences for a variety of bioinformatics analyses.

7. The genome information table provides data about genome sequences, their level of completion, number of replicons (chromosomes and plasmids), CDS, etc. The definitions of these terms are presented in table 1. Genome sequences have hierarchical organization. Contigs are the names given to the shortest components of the assembly, which are sequences taken from individuals. Contigs are assembled into longer scaffolds, and scaffolds are assembled into chromosomes if there is sufficient mapping information. Many genome assemblies have only been assembled to the scaffold level. Examine the table before answering the questions in the genome database table.

Table I. NCBI Genome Database: Definitions

Term	Definition	Application
BioSample	Biological sample name, description and identification number.	Provides information about the origin of the sample and its connection to sequencing projects.
BioProject	Sequencing project name, description and identification number.	Provides information about the project including details about the organism, sequencing statistics and research publications.
Assembly	Refers to how the nucleotide sequences of a genome have been put together. An assembly can represent a complete genome or a set of genome fragments which are not fully connected (contigs).	Informs users about the type of genome sequence available. Researchers need to be aware of whether they are working with a complete genome or a set of contigs.
Level	Refers to the level of completion of the genome; the dark circles indicate a complete genome. Circles with clear zones indicate genomes that are incomplete.	Informs users about the type of genome sequence available. Researchers need to be aware of whether they are working with a complete genome or a set of contigs.
Scaffolds	Refers to a series of contigs organized in the right order but not necessarily connected to each other. Complete genomes have one or very few scaffolds.	Informs users about the type of genome sequence available. Researchers need to be aware of whether they are working with a set of contigs, scaffolds or a complete genome.
Size	Indicates the size of the genome sequence.	Allows researchers to verify the identity of the organism of interest.
GC Content	Indicates the average % GC content of the genome sequence.	Allows researchers to verify the identity of the organism of interest.
CDS	Indicates the predicted number of protein coding genes.	Allows users to assess the number of proteins and the coding capacity of the genome.
FTP (R,G)	File transfer protocol. R= reference sequence file; G= GenBank file	Allows researchers to select the type of sequence file they want to download for bioinformatics analyses.
Release Date	Date in which the genome sequence was released.	Informs users about the dates of projects completion and release.

Questions for Exploring the NCBI Genome Database

1. How many *Escherichia coli* strain MG1655 genomes did your search retrieve?

2. How many of the *Escherichia coli* strain MG1655 genomes were reported as complete?

3. Select one of the complete genomes and document the following information:

    a. Record the accession numbers, we will use these numbers for the phage hunting part of this exercise.

    b. How many CDS does this genome have?

    c. What is the size and %GC content of the selected genome? How do these values compare to other strains of the same genera and species?

    d. Go to the FTP column and click on R or G. In the genome index page, select the file labeled as AssemblyNumber_genomic.fna.gz . Download and store the file in your computer, it contains the genome sequence of the *E.coli* strain selected.

4. Go to the assembly column and select one of the numbers listed. This action should take you to the assembly page. Write one or two sentences to briefly describe the information provided in this page.

5. Repeat steps 1-4 to investigate the genome sequences available for any of the bacteria we have studied in class. Choices include but are not limited to: *Staphylococcus epidermis, Pseudomonas putida, Rhodococcus erythropolis, Vibrio cholera, Bacillus anthracis, Bradyrhizobium japonicum, and Clostridium tetani.*

6. Organize the data obtained in steps 1-5 as a table that could be used to compare genome information for multiple bacterial species.

Part II. In Silico Phage Hunting with PHASTER

Using PHASTER to identify prophage regions within a bacterial chromosome. The following instructions may be used for instructor use to guide the lab activity.

1. Go to the website phaster.ca

2. Click the ‘RUN AN EXAMPLE’ tab on the bottom of the homepage to explore how PHASTER finds prophage regions in a bacterial genome. This will automatically generate a sample bacterial strain with prophage regions.

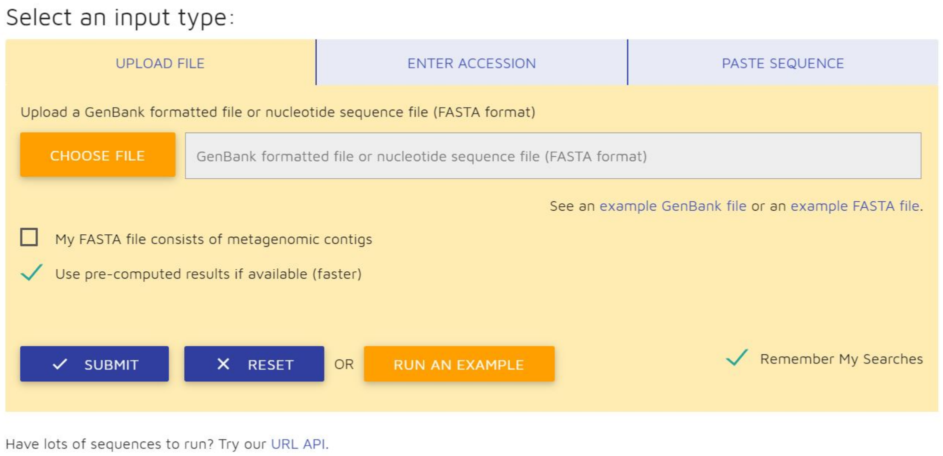

3. On the ‘SUMMARY’ page, view how many prophage regions PHASTER identified, marked as intact (green), questionable (blue), and incomplete (red). For this activity, only analyze the intact prophage regions, since these regions are marked as the most complete by the PHASTER algorithm.

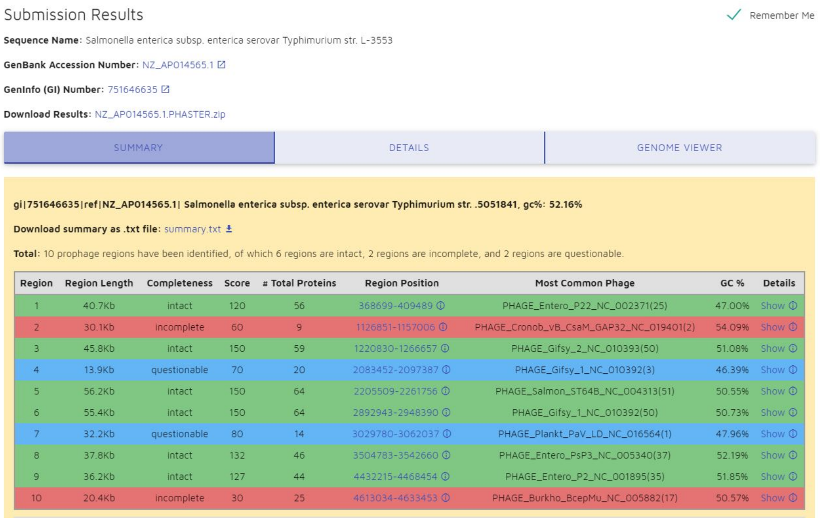

Submission Results

Remember Me

Sequence Name: Salmonella enterica subsp. enterica serovar Typhimurium str. L-3553

GenBank Accession Number: NZ_AP014565.1

GenInfo (GI) Number: 751646635

Download Results: NZ_AP014565.1.PHASTER.zip

SUMMARY | DETAILS | GENOME VIEWER

gi|751646635|ref|NZ_AP014565.1| Salmonella enterica subsp. enterica serovar Typhimurium str. .5051841, gc%: 52.16%

Download summary as .txt file: summary.txt

Total: 10 prophage regions have been identified, of which 6 regions are intact, 2 regions are incomplete, and 2 regions are questionable.

Region	Region Length	Completeness	Score	# Total Proteins	Region Position	Most Common Phage	GC %	Details
1	40.7Kb	intact	120	56	368699-409489	PHAGE_Entero_P22_NC_002371(25)	47.00%	Show
2	30.1Kb	incomplete	60	9	1126851-1157006	PHAGE_Cronob_vB_CsaM_GAP32_NC_019401(2)	54.09%	Show
3	45.8Kb	intact	150	59	1220830-1266657	PHAGE_Gifsy_2_NC_010393(50)	51.08%	Show
4	13.9Kb	questionable	70	20	2083452-2097387	PHAGE_Gifsy_1_NC_010392(3)	46.39%	Show
5	56.2Kb	intact	150	64	2205509-2261756	PHAGE_Salmon_ST64B_NC_004313(51)	50.55%	Show
6	55.4Kb	intact	150	64	2892943-2948390	PHAGE_Gifsy_1_NC_010392(50)	50.73%	Show
7	32.2Kb	questionable	80	14	3029780-3062037	PHAGE_Plankt_PaV_LD_NC_016564(1)	47.96%	Show
8	37.8Kb	intact	132	46	3504783-3542660	PHAGE_Entero_PsP3_NC_005340(37)	52.19%	Show
9	36.2Kb	intact	127	44	4432215-4468454	PHAGE_Entero_P2_NC_001895(35)	51.85%	Show
10	20.4Kb	incomplete	30	25	4613034-4633453	PHAGE_Burkho_BcepMu_NC_005882(17)	50.57%	Show

4. On the ‘SUMMARY’ page, click on the numbers shown in “Genome Position” column. This will automatically generate the genome sequence of the prophage region. Select the complete genome sequence of two -four intact prophage regions. Copy and paste the sequences into word or google docs. Save the files as a text, we will use these sequences for alignments and phylogeny next week.

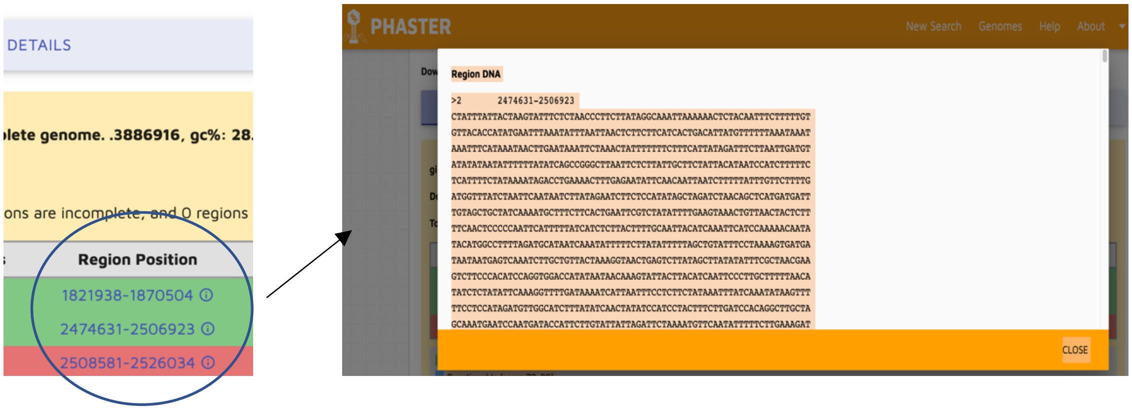

5. Click the ‘GENOME VIEWER’ tab to display the prophage regions as they appear on the bacterial chromosome

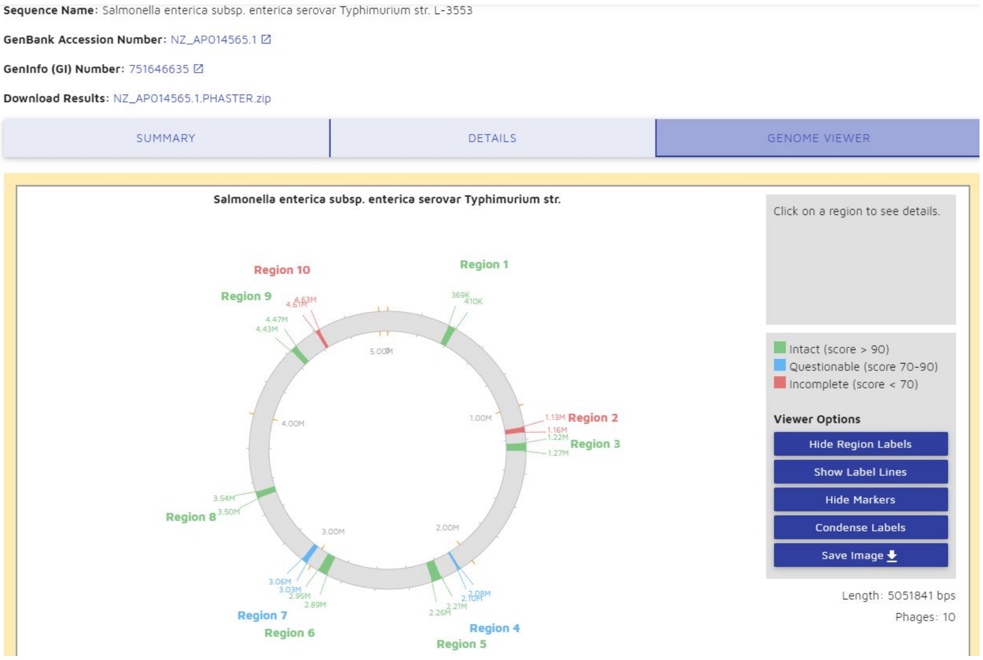

6. Click on an intact prophage region to view the subsequent proteins of the phage. Observe the key at the bottom which indicates the types of proteins present in the prophage region.

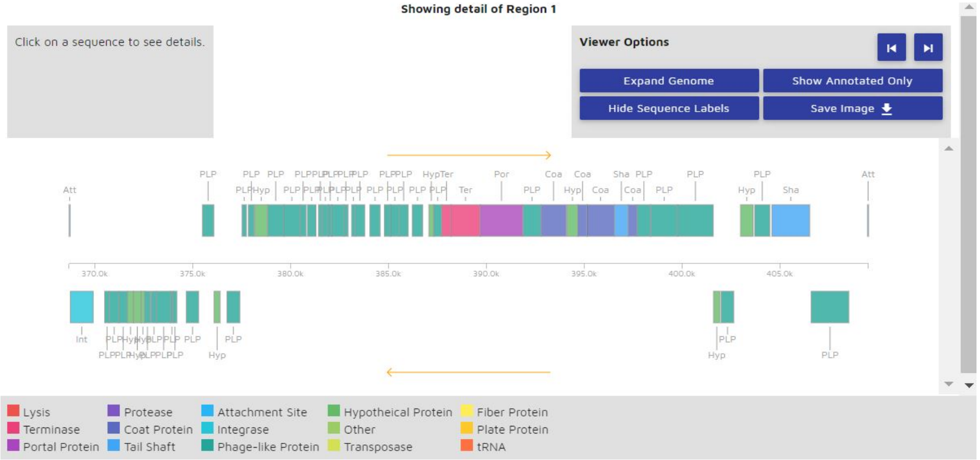

7. Many of the proteins that PHASTER identifies are hypothetical, meaning that this protein sequence has no known function. Other proteins, such as head and tail proteins, terminases, integrases, portal proteins, plate proteins, and others serve a distinct function for the bacteriophage. You will want to click the blue 'Show Annotated Only' button to view the annotated, or known-function, proteins in the prophage region.

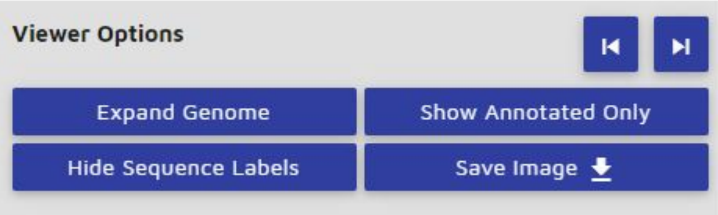

8. To practice, click the 'ENTER ACCESSION' tab on the homepage. Select an accession number from the bacterial genomes you investigated in part I (*Exploring the NCBI Genome Database*). Repeat steps 3-6 to analyze the prophage regions of each bacterial strain.

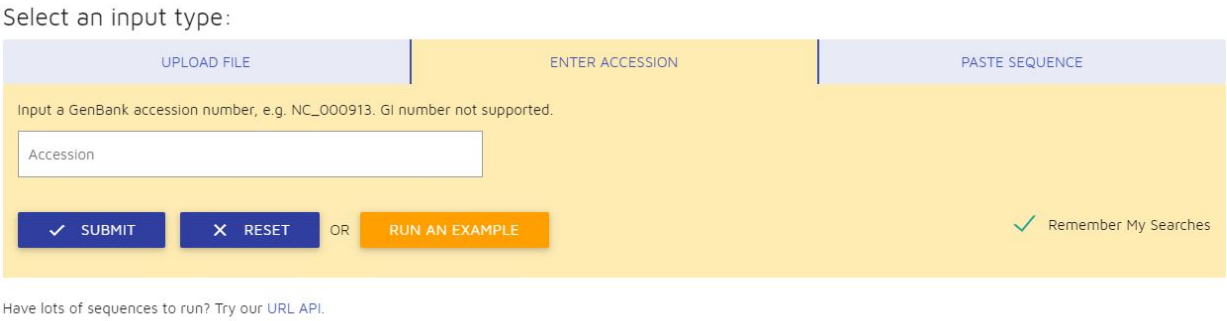

Questions for In Silico Phage Hunting with PHASTER

1) Examine the results generated by PHASTER. Click on the summary result file. Answer the following questions:

    a. How does PHASTER score phages as “intact”, “questionable” or “incomplete”? Briefly describe the criteria.
    b. How many intact phage genomes were identified in the bacterial genome sequence selected for analysis?
    c. What is the average length of the intact bacteriophages? How does it compare with size of the genome sequence selected for analysis?
    d. Which phages are listed in “Most Common Phage” column? What is the host organism for these viruses?

2) Continue to examine the results generated by PHASTER. Click on the “Details” column. Choose of one region designated as intact phage genome. Answer the following questions:

    a. Examine the CDS and BLAST Hits columns of the region selected. Do any of predicted CDSs encode proteins necessary for viral replication? Explain. Support your answer with at least two examples.

    b. Examine the E-value column. Based on the values shown, are the sequences in the CDS column likely to encode phage proteins? Explain. Support your answer with at least one example.

    c. Explore the BLAST Hit column for each intact prophage region. Locate the proteins annotated as “integrase”. Click “Show” in the “Sequence” column to get the protein sequence. Select the complete protein sequence of the integrases present in all the intact prophage regions. Copy and paste the sequences into word or google docs. Save the files as a text; these sequences will be used for alignments and phylogenetic analyses next week.

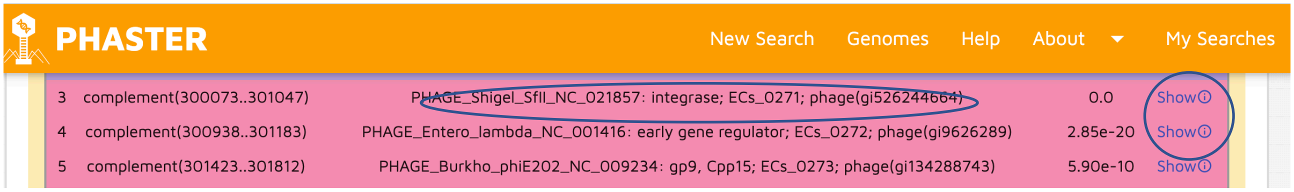

3) Click the 'GENOME VIEWER' tab to display the prophage regions as they appear on the bacterial chromosome. Click on an intact prophage region to view the subsequent proteins of the phage. Observe the key at the bottom which indicates the types of proteins present in the prophage region.

    a. Describe the organization and composition of the prophage genome. Which proteins of known function are shown in the diagram? What type of proteins are more common or abundant in the phage genome?

4) Select two proteins from the prophage region. Explain their relevance to the structure or replication of the virus.

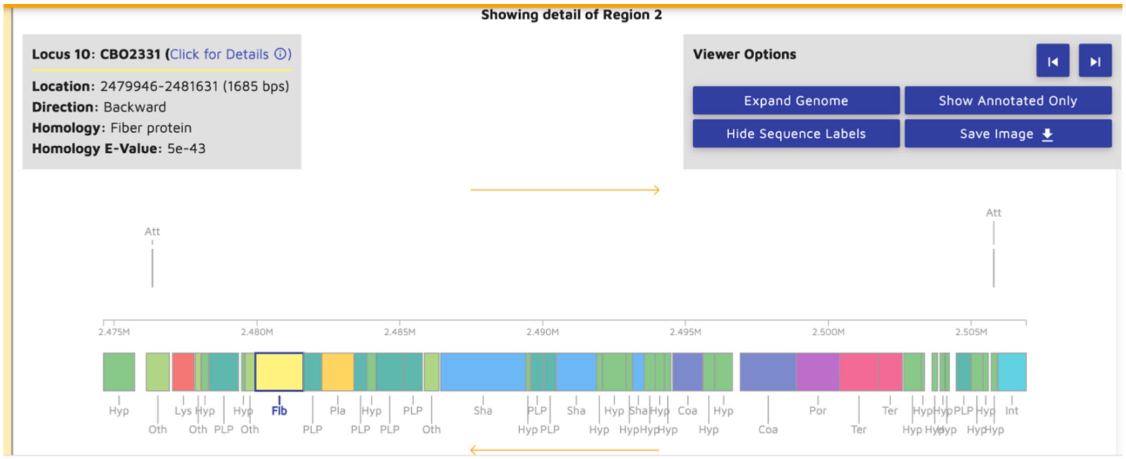

5) Discuss two ways the information generated by PHASTER analysis can help scientists design and conduct experiments to learn about bacteriophages?

In Silico-Phage Hunting
Worksheet III: Protein Sequence Alignments and Phylogenetic trees
Name: ________________________________

Objectives

- Construct and evaluate multiple protein sequence alignments
- Create phylogenetic trees using data from multiple sequence alignments
- Identify and interpret the basic information presented in phylogenetic trees
- Use phylogenetic trees to investigate the origins of an unknown protein sequence

Introduction

Scientists study evolutionary relationships amongst organisms by constructing phylogenetic trees. To build phylogenetic trees, we need to have a feature to compare amongst different organisms. A phylogenetic tree can be constructed using physical information (shape, bone structure, or behavior) or molecular information, like protein and DNA sequences.

Bacteriophages are smaller than most microbes (200 nms) and can only be visualized using electron microscopy. Viruses lack ribosomes, hence using ribosomal RNA to study evolutionary relationships amongst phages is not feasible. A common way to investigate phylogeny among diverse bacteriophages and prophages is by comparing viral protein sequences.

The goal of this activity is to construct protein sequence alignments and phylogenetic trees to investigate evolutionary relationships amongst bacteriophages. This exercise aims to examine protein sequences present in the bacteriophages you identified last week and explore whether they can be used to study phylogenetic relationships amongst these viruses. As a case study, we will investigate if viral integrases can be used to assess evolutionary relatedness amongst phages that infect different *E. coli* strains. Integrases are proteins that facilitate viral integration into the host's chromosome by recombination between a short sequence of phage DNA (*attP* site), and the bacterial attachment site (*attB*). Each integrase recognizes distinct DNA sequences. Based on this information, we hypothesize that integrases from phages that infect the same bacterial species will share a high level of similarity in their protein sequences. The procedure outlined below employs a variety of bioinformatics tools such as multiple sequence alignments and phylogenetic trees to address this question.

Multiple Sequence Alignments and Phylogenetic Trees

Multiple sequence alignments are an important first step for most methods of phylogenetic analysis and interpretation of evolutionary processes. In this exercise, we will use protein sequences retrieved while exploring the PHASTER tool last week. The Clustal omega or MAFFT programs will be used to create multiple sequence alignments of bacteriophage proteins. Subsequently, we will employ the programs' phylogeny functions to build phylogenetic trees and investigate the origin of an unknown phage integrase.

During the final part of this exercise we will explore the interactive Tree of Life (iTOL). This tool allows visualization, editing, and storage of phylogenetic tree data. You will use iTOL to create a publication-worthy picture illustrating the relationship between the "unknown" integrases and similar proteins found in the bacteriophage genomes.

Procedure

Part I. Multiple Protein Sequence Alignments with Clustal Omega

Note to Instructors: Clustal Omega can align up to 4000 sequences or maximum file size of 4MB. When data sets are larger than 4MB, I recommend using MAFFT. This program can accept up more than 50,000 sequences with efficient processing speed. MAFFT can be accessed at (https://mafft.cbrc.jp/alignment/server/). The activity described here was done using Clustal omega since it is user friendly and many students have used it in introductory courses.

1. Access Clustal Omega by using the following URL: https://www.ebi.ac.uk/Tools/msa/clustalo/. This bioinformatics tool allows users to align and compare protein sequences.

2. Locate the "test set" of genome sequences provided by the instructor. To do this, go to the class page in Canvas, click on the module labeled as "Phage Hunting". Select the folder labeled "PhageIntegrases_Set1". Download and save the file in your computer.

3. In the input window, click on "choose file" to upload the "PhageIntegrases_Set1" file containing the protein sequences for alignment.

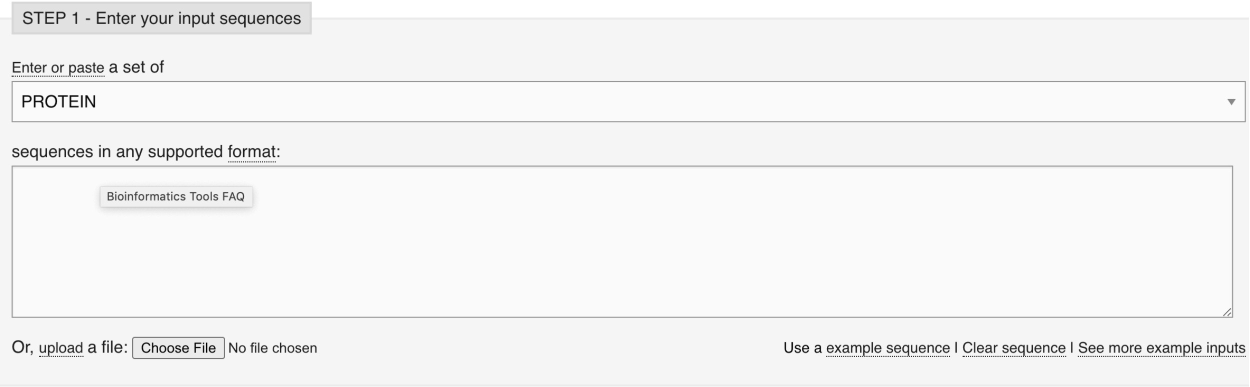

4. In the output format window, select ClustalW with characters counts. Click submit.

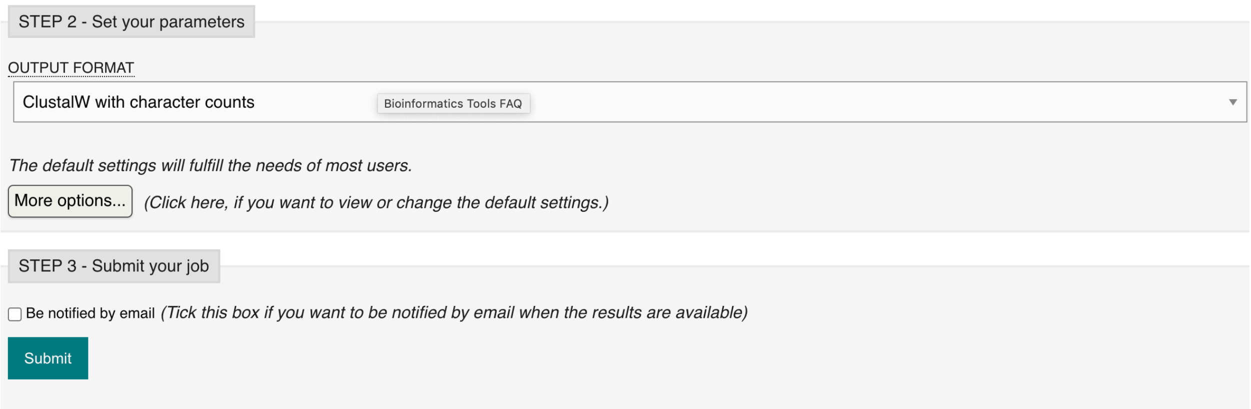

5. Examine the output of the alignment. On the alignment window select “show colors”. This tool allows us to easily visualize amino acids that are conserved amongst the aligned sequences. When examining alignments of different phage integrases note the amino acid residues that are identical (*), close homologs (:), and somewhat conserved (.).

6. Go to results summary and select “percent identity matrix”. Create a table to record the percent identity amongst the sequences analyzed (see example below).

Protein Seq ID	%identity to Seq 1	%identity to Seq 2	%identity to Seq 3	%identity to Seq 4	%identity to Seq 5
Seq 1	100	50	36	42	14
Seq2	32	100	65	88	34
Seq3	20	55	100	70	43
Seq4	18	45	77	100	25
Seq5	70	12	42	56	100

7. Go to results summary, then select “Phylogenetic Tree”. This tool will create a phylogenetic tree using the protein sequence alignment. Take a screen shot of the phylogenetic tree shown in the output window.

8. In the page showing the phylogenetic tree, choose “Download Phylogenetic Tree Data”. This command will download the phylogenetic tree file generated from the alignments. Copy and paste the tree data in a text editor or word processor. Save the file as plain text. This tree file will be used for further visualization and editing using the iTOL tool.

Part II. Using iTOL to Visualize and Edit Phylogenetic Trees

The following guide can be used by instructors for the purposes of visualizing, editing and organizing the phylogenetic trees constructed using prophage protein sequences obtained with PHASTER.

1. Visit the website https://itol.embl.de/

2. Go to "Create an account" to generate a username and password for iTOL. Having an account will also allow you to store and organize all the phylogenetic trees data files associated with your phage hunting project.

3. Select "Upload a tree". In this window, you can choose to upload the tree file you saved in part I or paste the tree data in the text window.

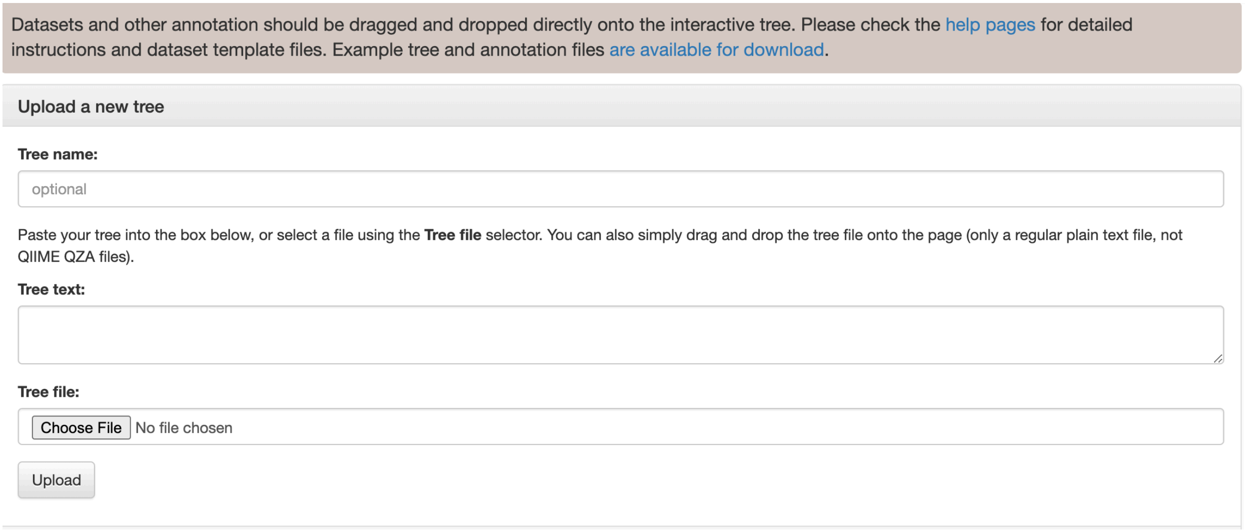

4. Examine the phylogenetic tree displayed in the iTOL window. Explore the control panel in the right-hand side of the page. Use the tools in the panel to edit and create a publication-worthy figure of your phylogenetic tree. The figure should include branch lengths, and highlight the clades, labels, and root of the tree. To practice, use the following editing commands:

    a. Click on display mode to view circular, normal and unrooted representations of the phylogenetic tree.
    b. Go to "Branch lengths" to explore how using or ignoring branch lengths changes the shape of the tree.

c. Select one of the tree nodes. Use your cursor to point at it and do a right click. This action will show the node functions and editing commands. Use the commands to edit the nodes and clades of the tree.

d. Once you have finished exploring the iTOL tools and editing your phylogenetic tree, click on the export tab. This action will allow you to export the tree image in variety of formats. Save the exported image in your project folder. The iTOL file manager automatically save a copy of the tree in your account.

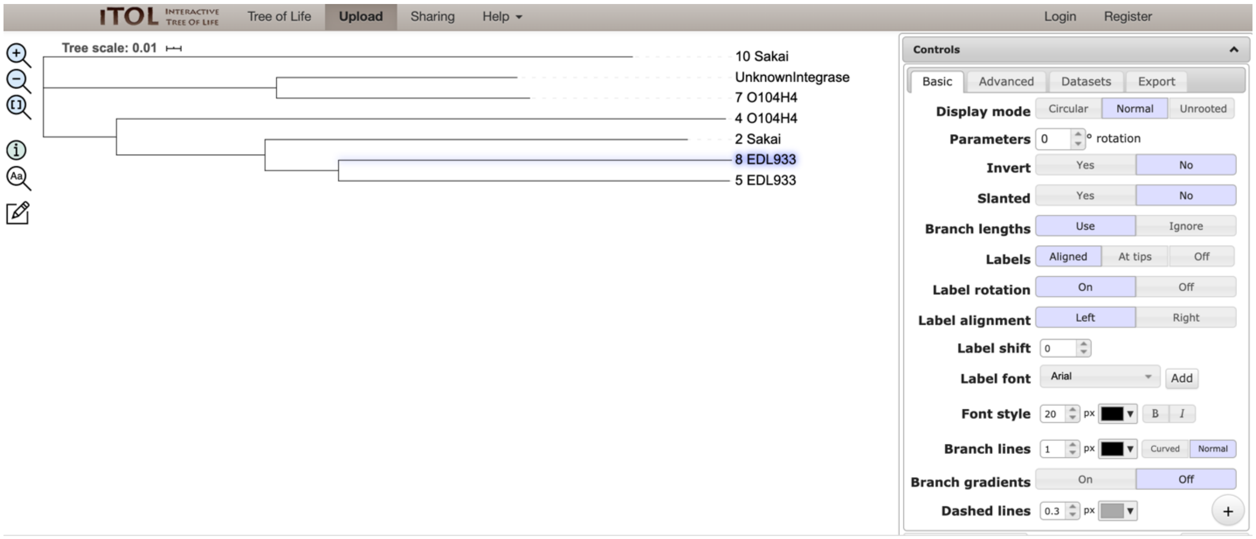

Questions for Multiple Sequence Alignments and Phylogenetic Trees

The set of protein sequences analyzed in this exercise belongs to prophages identified in pathogenic *Escherichia coli*, specifically enterohemorrhagic strains isolated during foodborne outbreaks in the USA (EHEC strain EDL933) and Germany (strain O104:H4). The data set also includes an unknown phage integrase associated with an *E. coli* strain linked to a recent outbreak. Answer the questions below to investigate which is closest homolog to the unknown phage integrase.

1. Examine the multiple sequence alignment generated by Clustal omega. Is the sequence alignment reliable? Explain.

2. List the amino acid residues that are identical or highly conserved amongst the integrase sequences aligned.   What amino acids are typically conserved in proteins with similar functions? Explain.

3. Based on the sequence alignment, which two sequences shared the highest level of sequence identity? Which sequences shared the lowest level of identity? What does a high % percent sequence identity suggest about the function of proteins? Support your answer with at least one example.

4. Examine phylogenetic trees created by Clustal omega and edited in iTOL.

    a. What is tree scale? What information does it provide?
    b. How many clades does the tree have? Highlight the clades with different colors.
    c. Circle the last common ancestor for the *E.coli* strain EDL933 integrases. Are there any other *E. coli* strains that share a common ancestor with the EDL933 strains? Explain.
    d. Does the phylogenetic tree show any sequences that can be considered outgroups? Explain.

5. According to the tree, which is the closest relative to the unknown phage integrase? Explain.

6. Based on the information presented in the phylogenetic tree, can you predict where the *E.coli* strain containing the unknown integrase came from? Explain.

7. Could the phage containing the “unknown” integrase infect other *E.coli* strains? Explain.

In Silico Phage Hunting
WS IV: Formulating Hypotheses and Designing an Experimental Plan
Name: ________________________

Objectives

- Search current scientific literature to learn about research on bacteriophages
- Formulate hypothesis about the abundance and diversity of phages in bacterial genomes
- Design an experimental plan to address the hypotheses posed using computational methods, or wet bench experiments

Introduction

During the past weeks, we have been working with several freely available bioinformatics tools to identify bacteriophages and explore their genomes. We have also learned that proteins present in phage genomes can be used to investigate phylogenetic relationship between these viruses. The next step logical step of the project is to formulate a research question and apply the skills you learned to address it.

This week, the laboratory exercise focuses on researching the scientific literature and thinking of research questions that could be addressed using bioinformatic methods, wet bench experiments or a combination of both. You must start by selecting a bacterial species that interest you, then you can investigate whether there is anything known about bacteriophages in that organism. Ideas for research projects can be related but are <u>NOT</u> limited to the following topics: abundance of phages amongst strains of the same bacterial species, investigating conservation and evolutionary relationships amongst viral proteins of phages that infect the same bacteria, presence of toxins, antibiotic resistance and catabolic genes in phage genomes, bioinformatic characterization of hypothetical proteins found phages, experiments to activate the lytic cycle of bacteriophages, etc.

Procedure

1. Go NCBI pubmed (http://www.ncbi.nlm.nih.gov/pubmed) and perform a literature search on bacteriophages and bacterial genera of your choice. List three of the references retrieved from the search. For each reference, list the authors' names, journal, year, and title of the papers. Write a paragraph summarizing the main findings of each paper.

    a. Ref1

    b. Ref2

    c. Ref3

2. State the research question and hypothesis for the experiment.

    a. Research Question

    b. Hypothesis

3. Briefly describe the rationale for the research question and the hypothesis stated above. Support your answer by referring to the papers listed in Question 1.

4. Describe the overall experimental approach that will be used to test the hypothesis of the project. Please include the bioinformatics tools to be used and describe the data you expect to obtain from these analyses.
