# Supplementary material for: In silico Phage Hunting: Bioinformatics Exercises to Identify and Explore Bacteriophage Genomes
Source: Front Microbiol. 2020 Sep 17;11:577634. doi: 10.3389/fmicb.2020.577634 (PMC7533560; doi:10.3389/fmicb.2020.577634)
Supplement: Supplementary file 9 [file Data_Sheet_9.PDF]

Bacteriophages

Bio5550: Microbiology

Hamline University

1

1

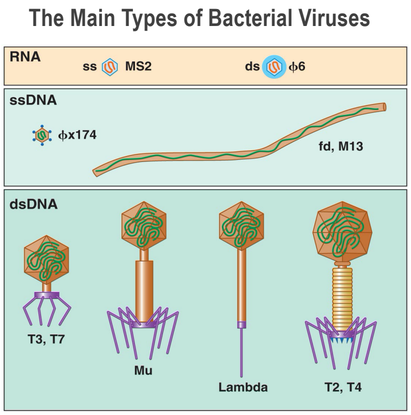

2

2

Overview of Bacterial Viruses

Why do we study bacteriophages?

- ✓ Bacteriophages are very diverse
- ✓ Best-studied bacteriophages infect enteric bacteria
  - ✓ E.g., *E. coli*, *Salmonella enterica*
- ✓ Most contain dsDNA genomes
- ✓ Most are naked, but some possess lipid envelopes
- ✓ They are structurally complex, containing heads and tails

3

3

Think/Pair/Share

- ✓ Describe the life cycle of a bacteriophage.

4

4

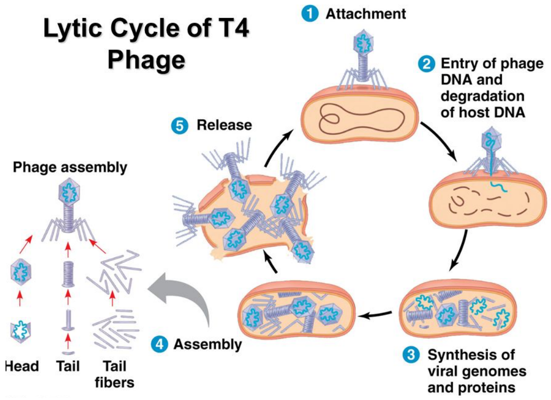

5

Bacteriophages Attach to Host Cells

- Contact and attachment are mediated by **cell-surface receptors**.
  - Proteins that are specific to the host species and which bind to a specific viral component.
  - Bacterial cell receptors are normally used for important functions for the host cell.
    - Example: sugar uptake

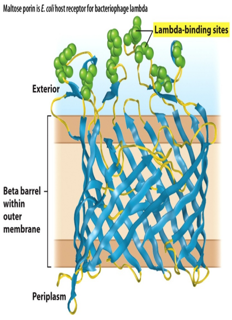

6

Phage Reproduction within Host Cells

- Most bacteriophages (phages) inject only their genome into a cell through the cell envelope.
  - The phage capsid remains outside, attached to the cell surface.
    - It is termed a "ghost."

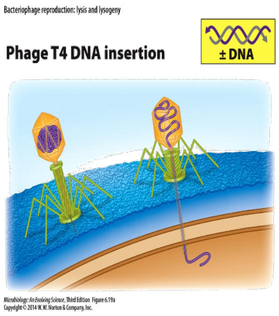

7

Attachment of Bacteriophage T4 to the Cell Wall of *E. coli*

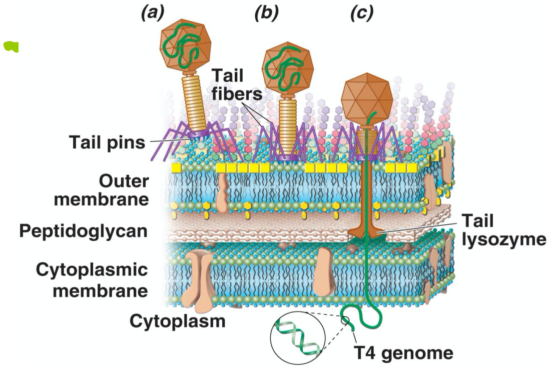

8

Viral Attachment and Penetration

✓ *Bacteriophage T4*: virus of *E. coli*; example of one of the most complex penetration mechanisms known

- ✓ Virions attach to cells via tail fibers that interact specifically with polysaccharides on *E. coli* cell envelope
- ✓ Tail fibers retract and tail core makes contact with *E. coli* cell wall
- ✓ Lysozyme-like enzyme forms small pore in peptidoglycan
- ✓ Tail sheath contracts and viral DNA passes into cytoplasm

9

9

Events in T4 Infection

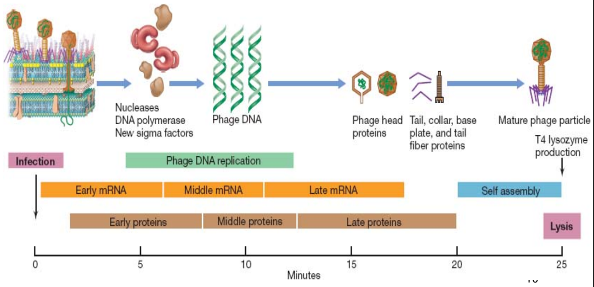

10

Production of Viral Nucleic Acid and Protein

✓ *Early proteins*

- ✓ synthesized soon after infection
- ✓ necessary for replication of virus nucleic acid

✓ *Late proteins*

- ✓ Synthesized later
- ✓ Include proteins of virus coat
- ✓ Typically structural components

11

11

Think/Pair/Share

Thinking like a Fred the Phage

✓ If you were a phage, what mechanism would you use to ensure that your genes, rather than those of the host, are transcribed ?

Give one example.

12

12

How do we know a phage has infected a bacterial population?

13

13

Quantification of Bacterial Virus by Plaque Assay

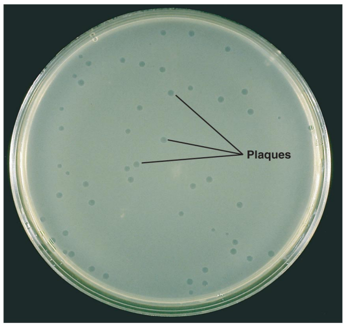

(b)

14

14

Cultivation and Isolation Viruses

- ✓ Infection of bacterial and animal cells with a particular virus is possible with modern cell culture techniques
- ✓ Viral infection and reproduction is visualized by the formation of plaques

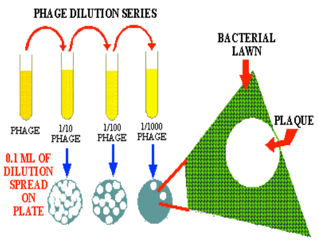

15

Temperate Bacteriophages

16

16

Temperate Bacteriophages: Lambda and P1

- ✓ *Temperate viruses*: can undergo a different life cycle resulting in a stable genetic relationship within the host
  - ✓ *Virulent mode*: viruses lyse host cells after infection
  - ✓ *Temperate mode*: viruses replicate their genomes in tandem with host genome and without killing host

17

17

The Consequences of Infection by a Temperate Phage

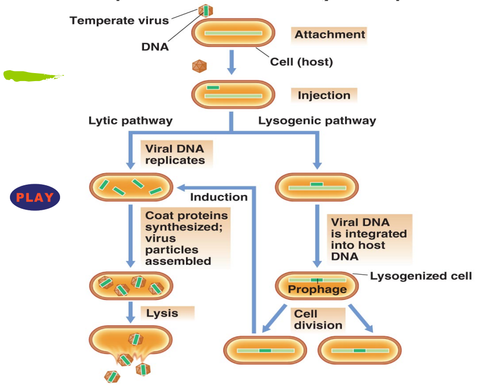

18

18

Temperate Viruses

- ✓ *Lysogeny*: state where most virus genes not expressed and virus genome (*prophage*) is replicated in synchrony with host chromosome
- ✓ *Lysogen*: a bacterium containing a prophage
- ✓ Under certain conditions lysogenic viruses may revert to the lytic pathway and begin to produce virions

19

19

Temperate Bacteriophages: Lambda, and P1

Bacteriophage Lambda

- ✓ Linear, dsDNA genome
- ✓ Complementary, single-stranded regions 12 nucleotides long at the 5′-terminus of each strand
- ✓ Upon penetration, DNA ends base-pair forming the *cos* site, DNA ligates and forms double-stranded circle
- ✓ When lysogenic, integrates into *E. coli* chromosome at the lambda attachment site (*att*λ)

20

20

Integration of Lambda DNA into the Host

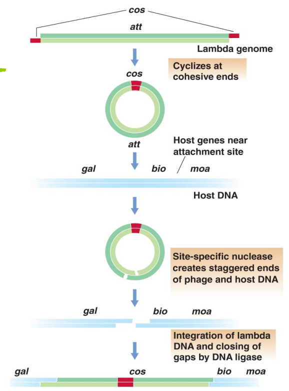

21

21

Think/Pair/Share

- ✓ If you were a phage, when would it be appropriate or advantageous to be integrated in the host's genome?
- ✓ When would be advantageous to propagate by lysis and kill the host?
- ✓ Why?

22

22

Take Home Message

- ✓ Bacteriophages attach to host cells using specific surface receptors
- ✓ Bacteriophages can propagate in permissive cells because shortly after infection, the virus take over the cellular machinery and modifies it to recognize and propagate its own genome
- ✓ A host is needed to isolate a virus; plaque assays are commonly used for phage isolation
- ✓ Bacteriophages can undergo lytic or a lysogenic cycles depending on cellular conditions

23

23

Bacteriophages

Bio5550: Microbiology

Hamline University

1

This presentation was created to provide an overview of the properties and the life cycle of bacteriophages.

Start by asking the class the following questions:

Have you ever heard the word bacteriophage?

What are Bacteriophages? Which organisms are hosts for these viruses?

The Main Types of Bacterial Viruses

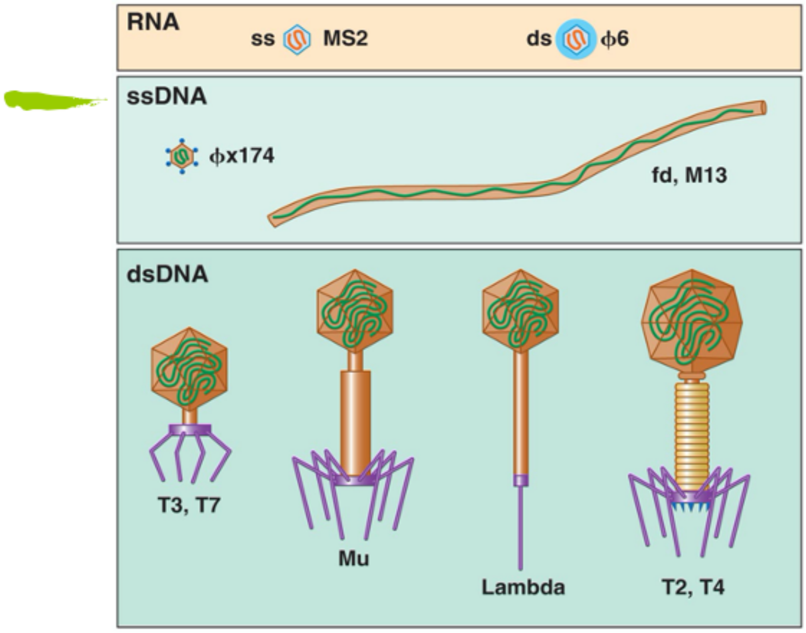

2

Use the diagram illustrated in the figure to start a discussion about phages. Ask the following questions:

What type of genetic material do phages contain?

Can these viruses be classified based on the genetic material they contain?

What are the main differences amongst the phages illustrated in this figure?

What is common to all phages shown in this diagram?

Explain the basic structure of the phage: Head, tail, fibers, neck, etc.

Can you compare the phages in shown in this figure to other viruses you have studied before?

Overview of Bacterial Viruses

Why do we study bacteriophages?

- ✓ Bacteriophages are very diverse
- ✓ Best-studied bacteriophages infect enteric bacteria
  - ✓ E.g., *E. coli*, *Salmonella enterica*
- ✓ Most contain dsDNA genomes
- ✓ Most are naked, but some possess lipid envelopes
- ✓ They are structurally complex, containing heads and tails

3

Use the slide to wrap up the discussion about phages and their comparison to other viruses. It is important to emphasize that phages only infect bacteria, not animal cells.

In this slide, the instructor can refer to biotechnological applications of phages in industry and medicine. For example, phage therapy for antimicrobial resistant infections and treating fruits and vegetables with phage cocktails to prevent foodborne outbreaks caused by Salmonella and Listeria. This is a great opportunity to show additional slides, illustrating the difference between enveloped and naked viruses.

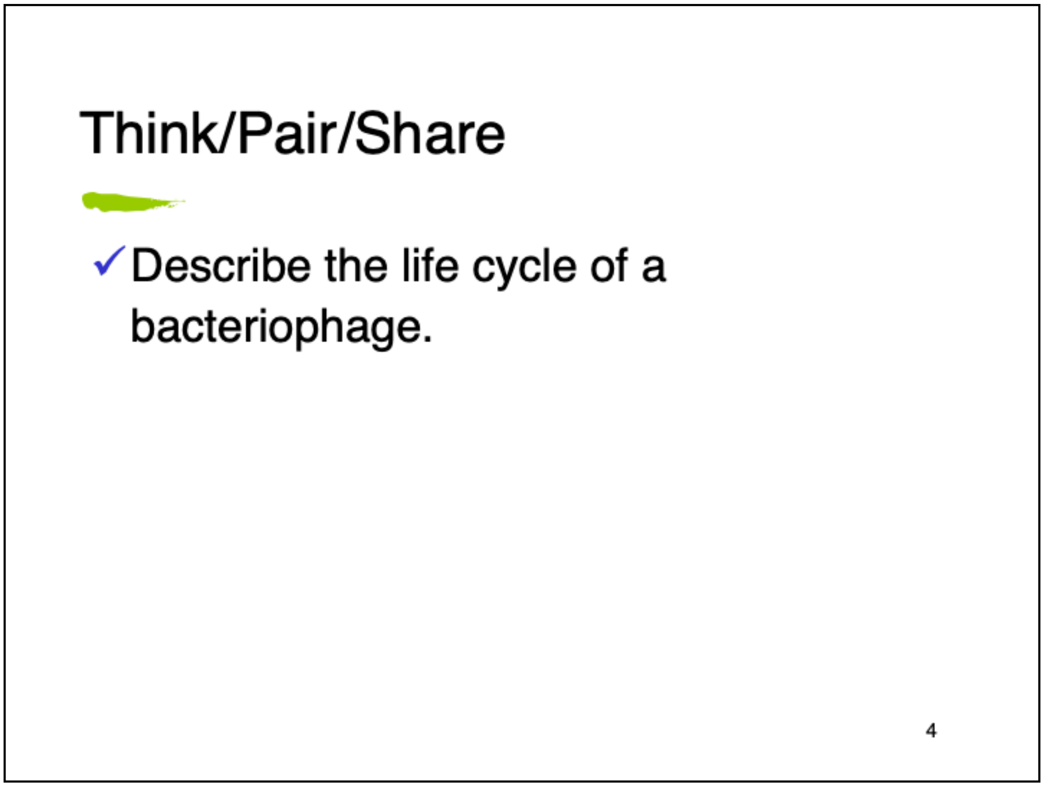

Students taking this class have learned a little bit about viruses in introductory biology courses and genetics. Ask them to propose a life cycle for the bacteriophage based on what they know about the life cycle of other viruses. I expect students to mention general steps such as host recognition, entry, and exit. Many times they forget replication of the genetic material is essential to reproduction.

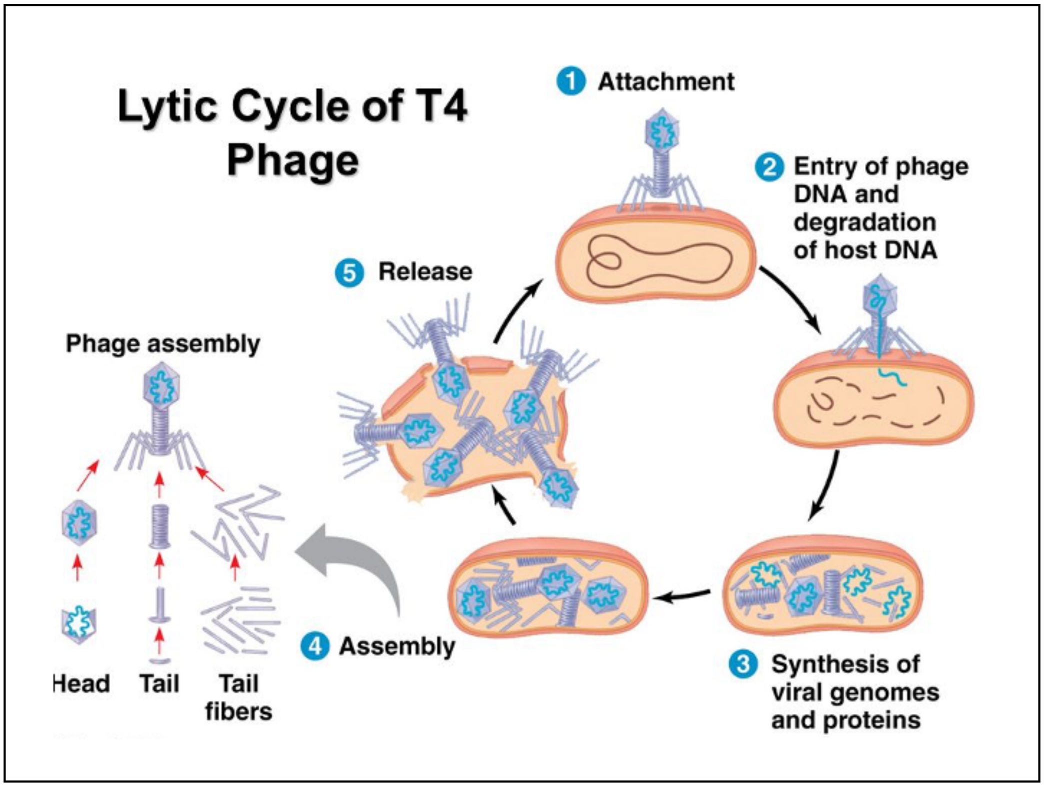

Explain the life cycle illustrated in the figure in detail. This is an opportunity for the instructor to talk about which event during the life cycle is most critical for successful reproduction. This is also a great opportunity to highlight that phages are very specific and can recognize only certain receptors in the host. It is a good opportunity to compare them to eukaryotic viruses and their tropisms, as well. This discussion leads right into the next slide about attachment to the host.

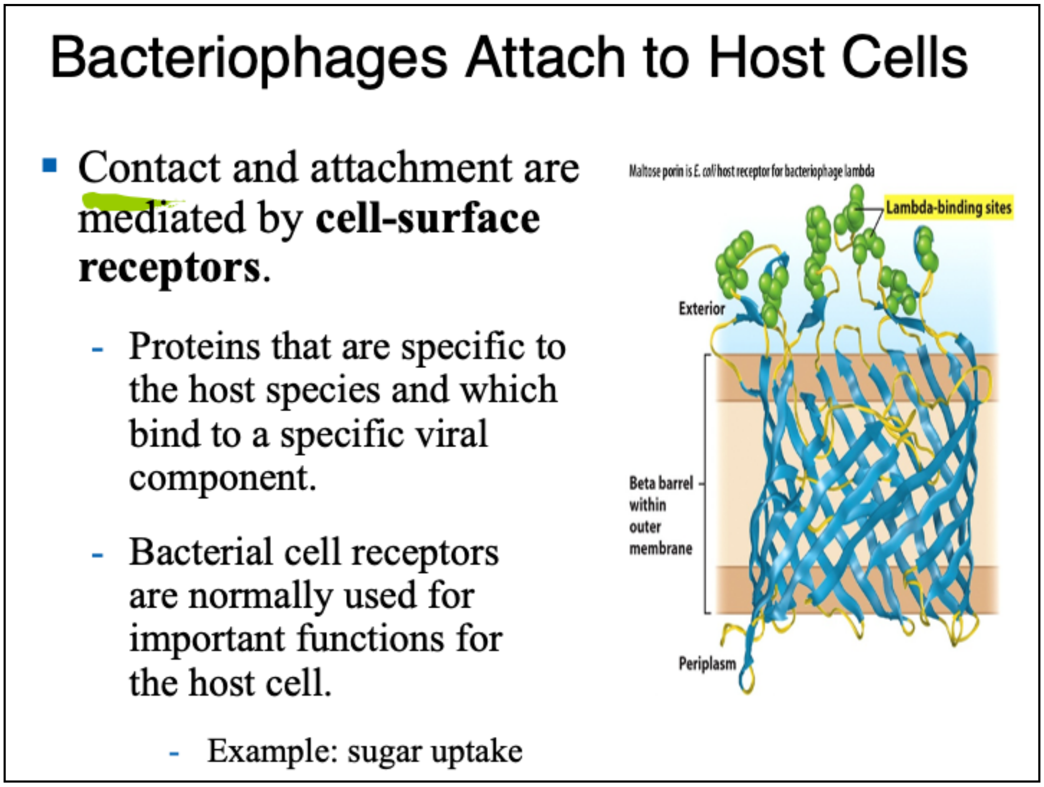

This slide is very appropriate to continue the discussion about viral recognition of receptors in host cells. The instructor should describe the porin and refer to its location in the outer membrane. Then, talk about the binding site where the phage lambda attaches.

Figure 6.18 Maltose porin is *E. coli* host receptor for bacteriophage lambda. The “beta barrel” of maltose porin (blue) is buried in the outer membrane. The phage-binding sites (green) were identified by amino acid substitution mutations that prevent phage binding and confer host resistance to lambda (PDB code: 1MAL).

Phage Reproduction within Host Cells

- Most bacteriophages (phages) inject only their genome into a cell through the cell envelope.
  - The phage capsid remains outside, attached to the cell surface.
    - It is termed a "ghost."

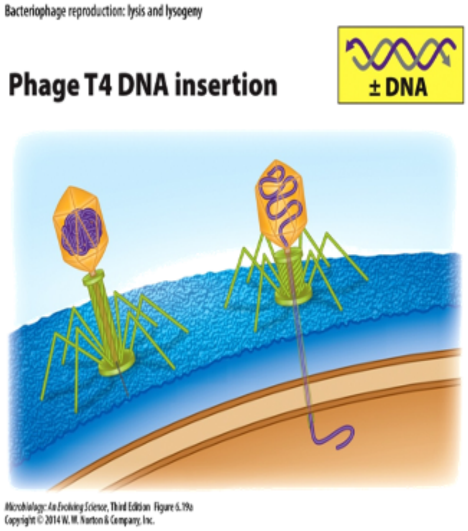

This slide is helpful to continue the discussion on host recognition and move to the next step of the cycle, injection of genetic material. As an instructor, I emphasize that phages mostly contain double stranded DNA in their genomes. They do not enter the host but effectively deliver the DNA to the cytoplasm for replication . The empty phage capsid is a known as " a ghost". This is a good point to ask students ' "what do you think happens to the phage's DNA after injection".

Figure 6.19A Bacteriophage reproduction: lysis and lysogeny. Phage T4 attaches to the cell surface by its tail fibers and then contracts to inject its DNA. The empty capsid remains outside as a "ghost."

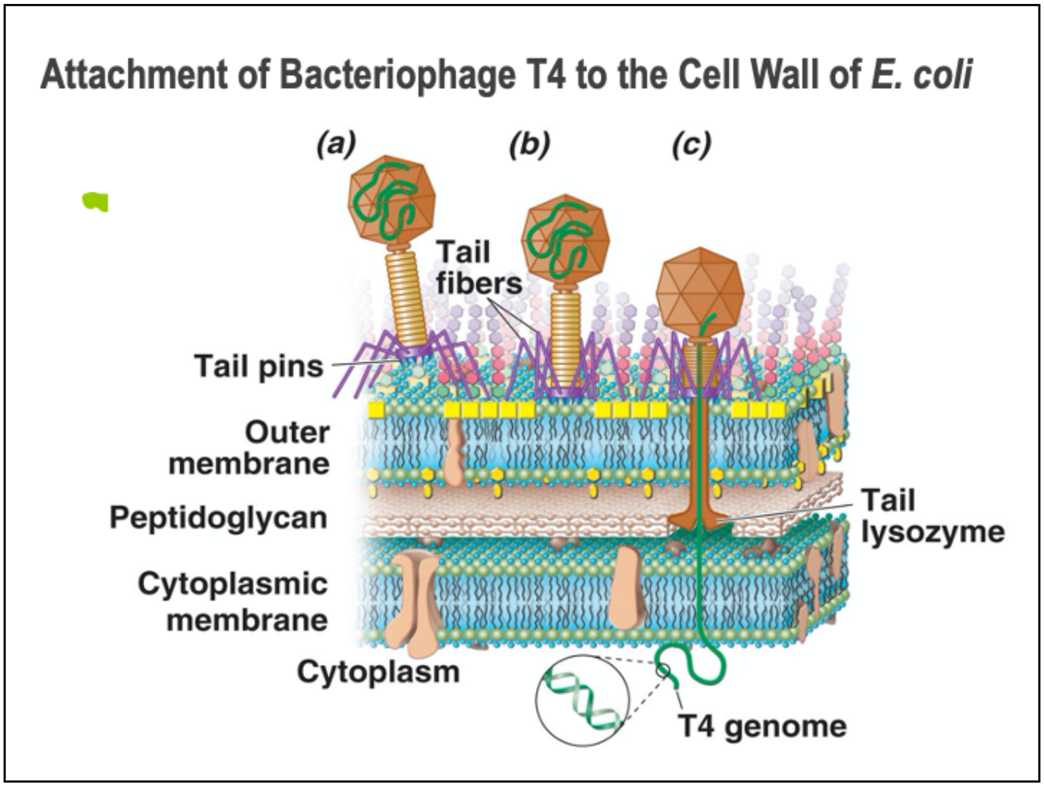

This slide continues the conversation about phage attachment and injection of genetic material into the host cells. Instructors have the opportunity to use the figure to review the basic features of Gram negative cell walls (highlight the outer membrane, LPS, thin peptidoglycan, and membrane proteins). This diagram is also a good tool to talk about tail pins and tail fibers and their role in attachment to the host cells. These tail structures mediate and secure attachment of the phage to LPS and secure entry of the phage's DNA into the cell.

Viral Attachment and Penetration

- ***Bacteriophage T4***: virus of *E. coli*; example of one of the most complex penetration mechanisms known
  - Virions attach to cells via tail fibers that interact specifically with polysaccharides on *E. coli* cell envelope
  - Tail fibers retract and tail core makes contact with *E. coli* cell wall
  - Lysozyme-like enzyme forms small pore in peptidoglycan
  - Tail sheath contracts and viral DNA passes into cytoplasm

9

This slide is an excellent tool to follow the discussion about tail fibers and process host entry. I ask students to think about lysozyme and its role in other systems.

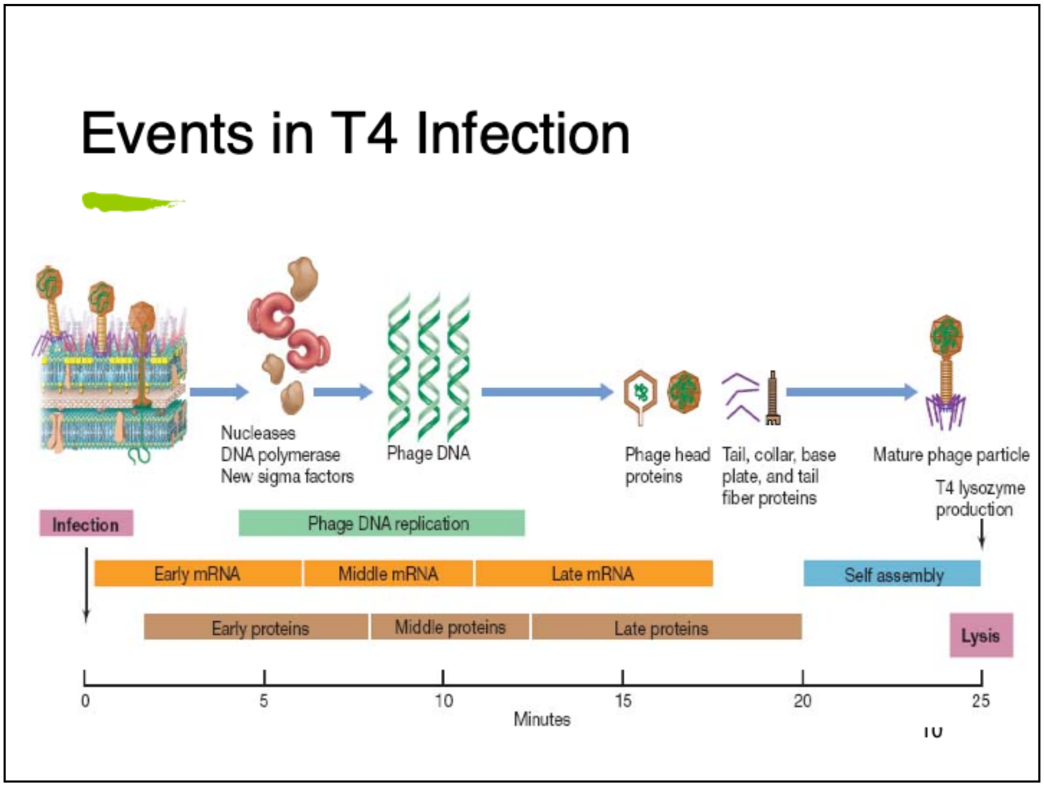

This slide presents a detailed description of the events happening at the molecular level during T4 phage infection. The instructor should highlight the role of early, middle and late proteins in phage replication. It is also a good opportunity to ask students how they think the phage gets to express its own genes once inside the host. This is a good time to start thinking about potential mechanisms to highjack the host's replication machinery. I also suggest the instructors ask students to match what is present in this diagram to the stages of infection present in slide 5.

Production of Viral Nucleic Acid and Protein

- *Early proteins*
  - synthesized soon after infection
  - necessary for replication of virus nucleic acid
- *Late proteins*
  - Synthesized later
  - Include proteins of virus coat
  - Typically structural components

11

This slide is shown to support and continue the discussion started in slide #10.

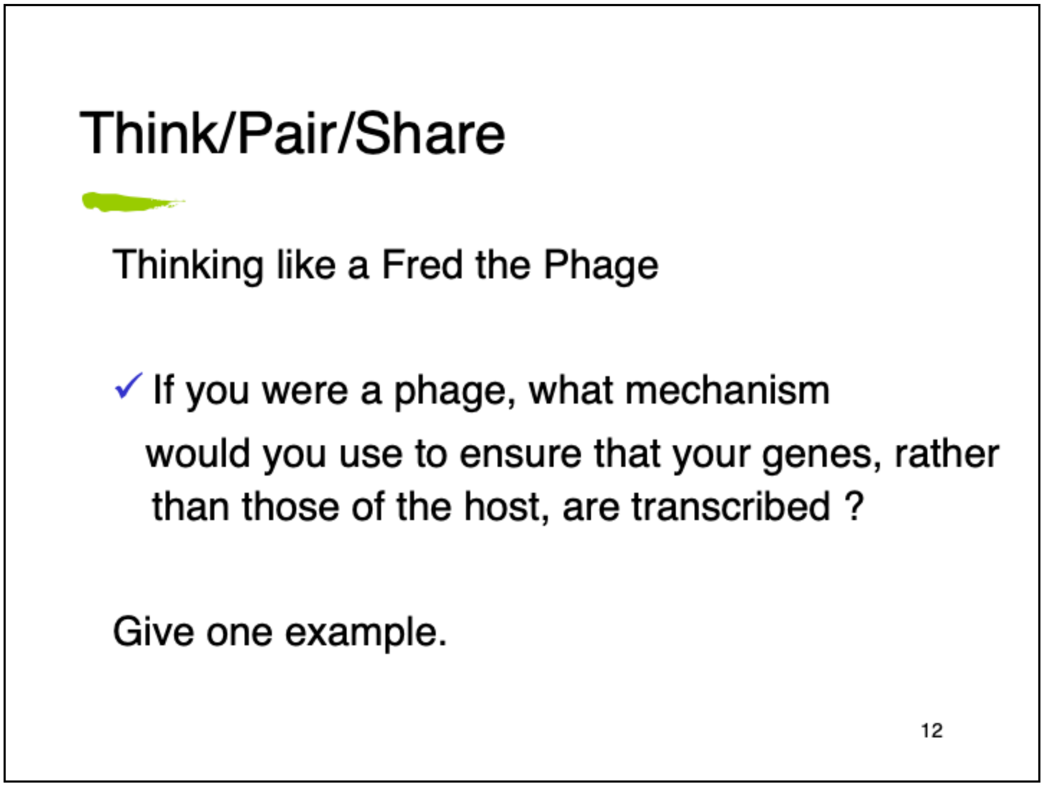

This THINK/PAIR/SHARE activity brings together the concepts discussed in slides 8-10. It provides an excellent opportunity for students to combine their newly acquired knowledge of phage infection together with genetics and molecular biology concepts. Encourage them to apply concepts from prior courses to make an educated guess of how phages trick the host's replication machinery to reproduce.

Answers might include but are not limited to: Modification of RNA Pol, aid in promoter recognition, nucleic acid modification, Phage sigma factor replace sigma70, covalent modification of RNAPol

How do we know a phage has infected a bacterial population?

13

Quantification of Bacterial Virus by Plaque Assay

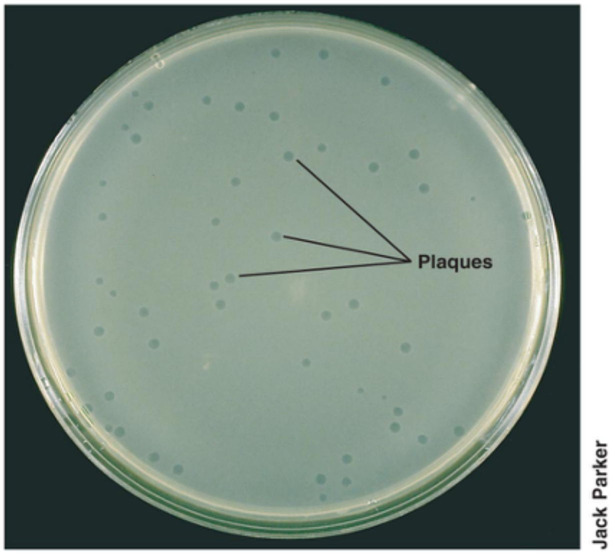

(b)

14

This slide introduces the concept of a plaque. The instructor should ask students to observe the picture and discuss what they see. Then, ask the following questions: Why do we look for plaque to detect phage infection? Where do the plaques come from? What do plaques illustrate? How can we use plaques to quantify phage infections? This last question leads right into the next slide, cultivation and isolation of viruses.

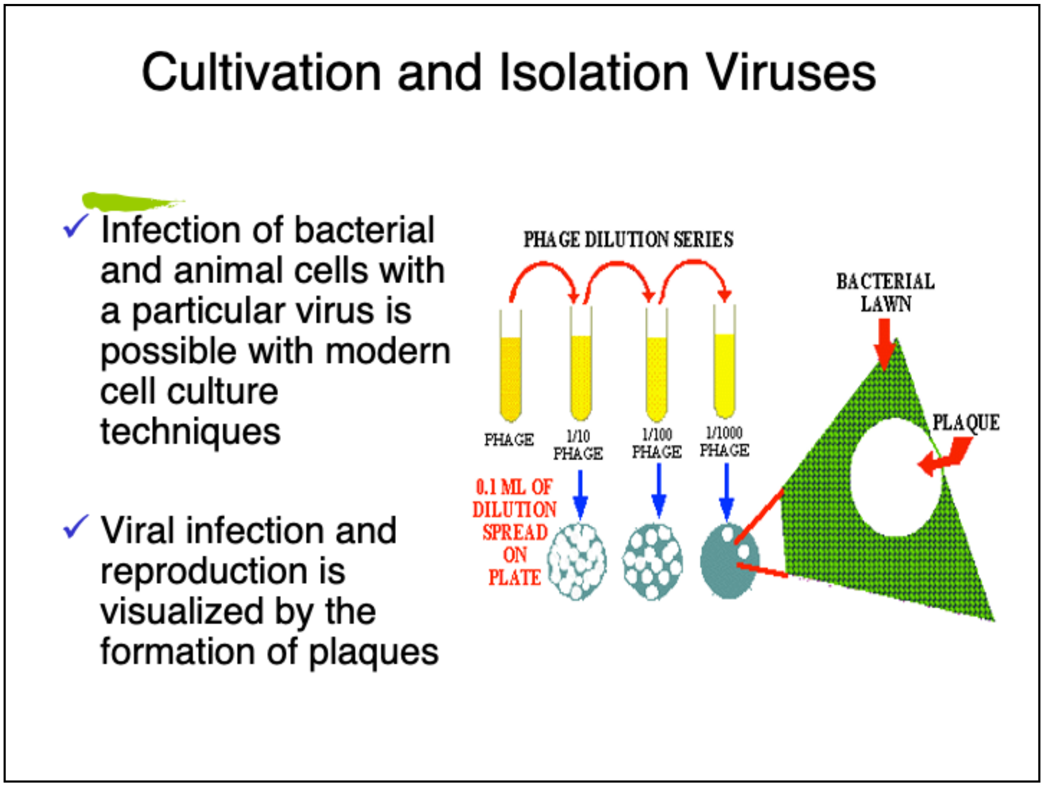

The instructor should explain the diagram. I suggest you compare it to a process students are familiar with like serial dilutions to perform microbial plate counts. Explain the formation of plaques and the appearance of them in a bacterial cell lawn. This is also a good time to ask students the following questions:

Do all plaques look the same?

How would infection by a defective phage look?

This a good topping point for the lecture. You can introduce and discuss temperate phages after a break or on a different lecture day.

Temperate Bacteriophages

16

Start the lecture by reviewing the previous discussion on phages. Mention that lysis of host cells is a result of phage infection. Then, ask: What would happen if a phage cannot lyse its host? Students' answers might include but are not limited to: phage death, cells death by mechanism that do not cause lysis, etc.

Follow up with these questions: Is it advantageous for a phage to always kill its host? Why? Time for discussion.

Temperate Bacteriophages: Lambda and P1

- ✓ *Temperate viruses*: can undergo a different life cycle resulting in a stable genetic relationship within the host
  - ✓ *Virulent mode*: viruses lyse host cells after infection
  - ✓ *Temperate mode*: viruses replicate their genomes in tandem with host genome and without killing host

17

Introduce the concept of temperate phages. Connect this concept with the benefits of not killing the hosts discussed during the Think/Pair/Share

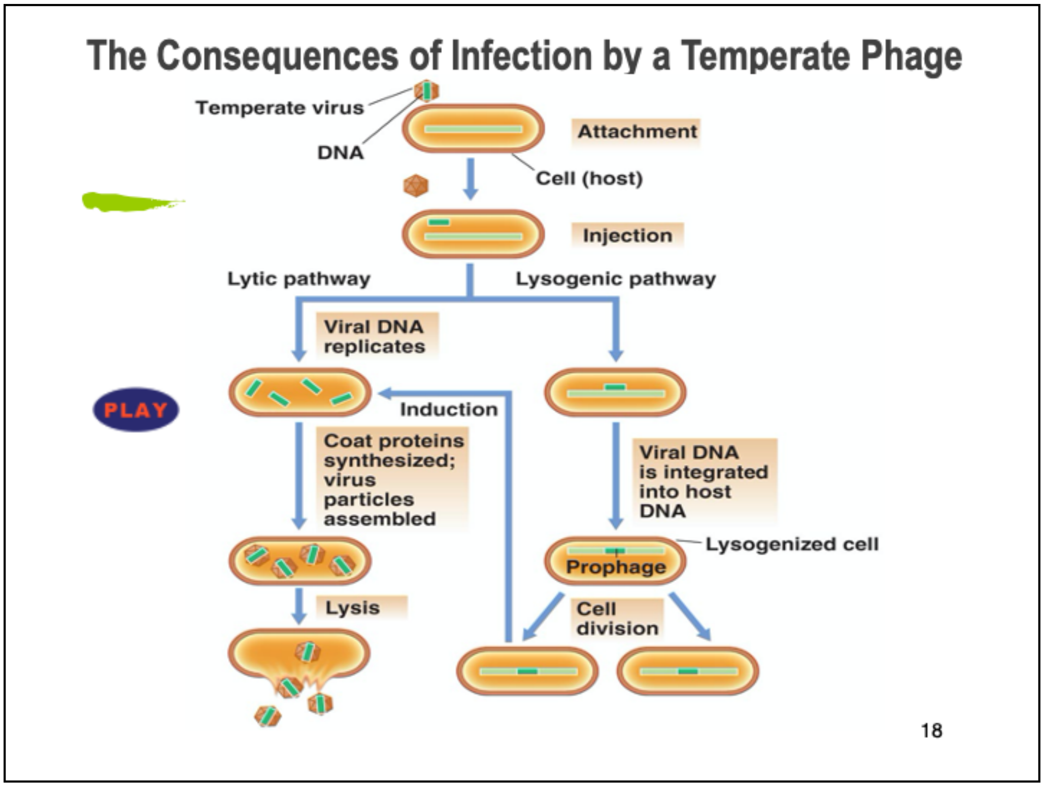

Use this slide to explain the differences between the lytic and lysogenic cycles in detail. You can also use a movie or animation to support the explanation of the cycles. Ask students about the differences and similarities between the cycles. You could also start encouraging students to think about what triggers one cycle vs. another.

Temperate Viruses

- ✓ *Lysogeny*: state where most virus genes not expressed and virus genome (*prophage*) is replicated in synchrony with host chromosome
- ✓ *Lysogen*: a bacterium containing a prophage
- ✓ Under certain conditions lysogenic viruses may revert to the lytic pathway and begin to produce virions

19

This slide aims to teach students the jargon used by microbiologists when discussing temperate phages. The instructor should emphasize the differences between the words lysogen and lysogeny. Then continue the discussion by asking students the following question: If you were a phage, what would make you kill your host?

Temperate Bacteriophages: Lambda, and P1

Bacteriophage Lambda

- ✓ Linear, dsDNA genome
- ✓ Complementary, single-stranded regions 12 nucleotides long at the 5′-terminus of each strand
- ✓ Upon penetration, DNA ends base-pair forming the *cos* site, DNA ligates and forms double-stranded circle
- ✓ When lysogenic, integrates into *E. coli* chromosome at the lambda attachment site (*att*λ)

20

This slide is optional. The instructor might use it to provide examples of temperate phages and their genomes and proteins. The description of the genome and cos site leads right into the next slide, which discusses the process of integration in the genome. Alternatively, the instructor might decide that the mechanism of phage integration is beyond the scope of the course. In this case, skip slides 20 and 21 and end the lecture with the Think/Pair/Share in slide #23.

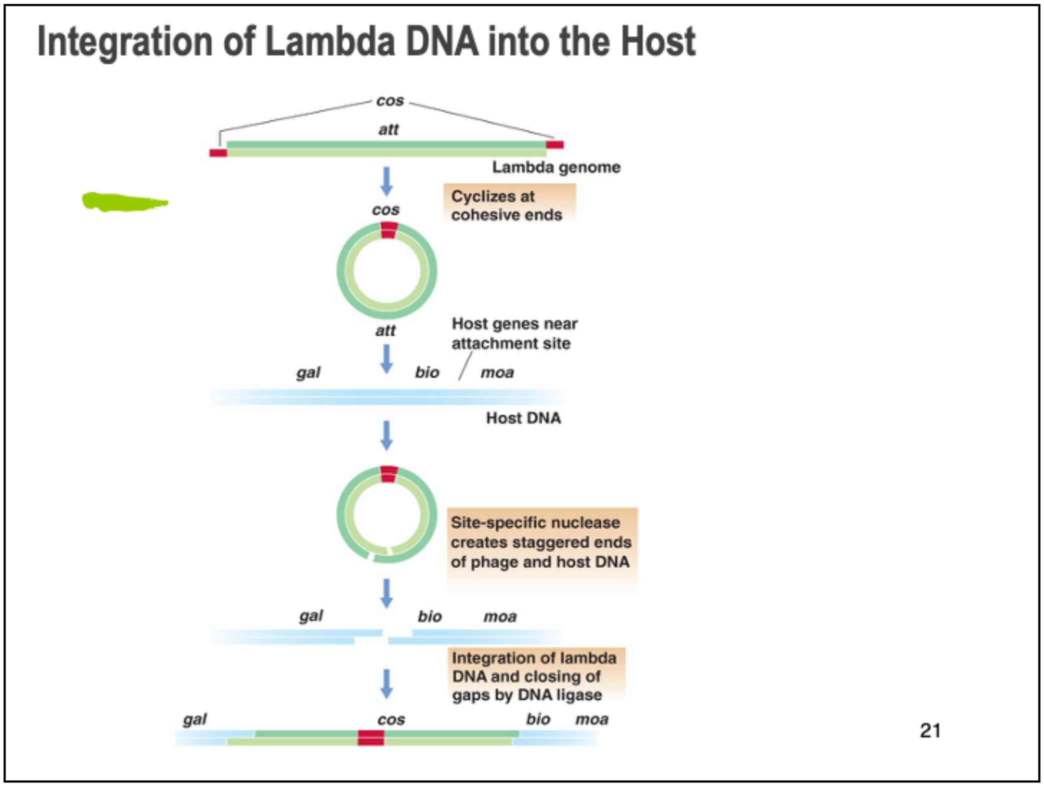

This slide is optional. The instructor might use it to provide examples of temperate phages and their genomes and proteins. The description of the genome and cos site complements the previous slide and allows students to visualize the process of integration in the host's genome. Alternatively, the instructor might decide the mechanism of phage integration is beyond the scope of the course. In this case, skip slides 20 and 21 and end the lecture with the Think/Pair/Share in slide #23.

Think/Pair/Share

- ✓ If you were a phage, when would it be appropriate or advantageous to be integrated in the host's genome?
- ✓ When would be advantageous to propagate by lysis and kill the host?
- ✓ Why?

22

Discuss this question with the class. What happens to the phage if all of its hosts are dead?

Take Home Message

- ✓ Bacteriophages attach to host cells using specific surface receptors
- ✓ Bacteriophages can propagate in permissive cells because shortly after infection, the virus take over the cellular machinery and modifies it to recognize and propagate its own genome
- ✓ A host is needed to isolate a virus; plaque assays are commonly used for phage isolation
- ✓ Bacteriophages can undergo lytic or a lysogenic cycles depending on cellular conditions

23

The instructor can decide whether a given class can continue learning about the molecular mechanisms mediating the decision between lysis and lysogeny. Instructors could potentially do another lecture about the role of Cro, CI, and CII in the phage’s decision to undergo lysis or lysogeny .
